# Plasticity between MyoC- and MyoA-Glideosomes: An Example of Functional Compensation in *Toxoplasma gondii* Invasion

**DOI:** 10.1371/journal.ppat.1004504

**Published:** 2014-11-13

**Authors:** Karine Frénal, Jean-Baptiste Marq, Damien Jacot, Valérie Polonais, Dominique Soldati-Favre

**Affiliations:** Department of Microbiology and Molecular Medicine, CMU, University of Geneva, Geneva, Switzerland; University of Heidelberg Medical School, Germany

## Abstract

The glideosome is an actomyosin-based machinery that powers motility in Apicomplexa and participates in host cell invasion and egress from infected cells. The central component of the glideosome, myosin A (MyoA), is a motor recruited at the pellicle by the acylated gliding-associated protein GAP45. In *Toxoplasma gondii*, GAP45 also contributes to the cohesion of the pellicle, composed of the inner membrane complex (IMC) and the plasma membrane, during motor traction. GAP70 was previously identified as a paralog of GAP45 that is tailored to recruit MyoA at the apical cap in the coccidian subgroup of the Apicomplexa. A third member of this family, GAP80, is demonstrated here to assemble a new glideosome, which recruits the class XIV myosin C (MyoC) at the basal polar ring. MyoC shares the same myosin light chains as MyoA and also interacts with the integral IMC proteins GAP50 and GAP40. Moreover, a central component of this complex, the IMC-associated protein 1 (IAP1), acts as the key determinant for the restricted localization of MyoC to the posterior pole. Deletion of specific components of the MyoC-glideosome underscores the installation of compensatory mechanisms with components of the MyoA-glideosome. Conversely, removal of MyoA leads to the relocalization of MyoC along the pellicle and at the apical cap that accounts for residual invasion. The two glideosomes exhibit a considerable level of plasticity to ensure parasite survival.

## Author Summary

*Toxoplasma gondii* can infect most warm-blooded animals, and is an important opportunistic pathogen for humans. This obligate intracellular parasite is able to invade virtually all nucleated cells, and as with most parasites of the Apicomplexa phylum, relies on a substrate-dependent gliding motility to actively penetrate into host cells and egress from infected cells. The conserved molecular machine (named glideosome) powering motility is located at the periphery of the parasite and involves the molecular motor, myosin A (MyoA). The glideosome exists in three flavors, exhibiting the same overall organization and sharing some common components while being spatially restricted to the central IMC, the apical cap and the basal pole of the parasite, respectively. The central and apical glideosomes are associated with MyoA (MyoA-glideosome) whereas the basal complex recruits myosin C (MyoC). Deleting components of the MyoC-glideosome uncovers the existence of complementary and compensatory mechanisms that ensure successful establishment of infection. This study highlights a higher degree of complexity and plasticity of the gliding machinery.

## Introduction

The phylum of Apicomplexa groups numerous important animal and human pathogens. The best-studied members include the medically important *Plasmodium* species and *Toxoplasma gondii* for which robust reverse genetic approaches have been developed. *T. gondii* belongs to the subgroup of Coccidians that comprises other cyst-forming parasites such as *Neospora*, *Eimeria*, *Cryptosporidium* and *Sarcocystis* species that infect the intestinal tracts of animals and cause foodborne diseases referred to as coccidioses. In humans, *Cryptosporidium* can cause enteritis while *T. gondii* infection is usually asymptomatic or causes flu-like symptoms. Up to one third of the world's population is infected by *T. gondii*, generally without consequence because the immune response constrains the parasite to persist as a dormant encysted form. However, this form lasts for the life span of its host and can reactivate into an invasive and fast-replicating stage in case of immunosuppression [1].

Apicomplexan parasites are surrounded by a three-layered pellicle composed of a classical external plasma membrane (PM) and a double-membranous inner membrane complex (IMC) constituted of flattened vesicles [2]. Freeze fracture analyses of the pellicle of *Toxoplasma*, *Eimeria* and *Sarcocystis* have revealed a structural compartmentalization of the IMC with a cone-shaped plate called apical cap, and the remainder regularly arranged in longitudinal strips joined at the posterior pole [3], [4]. More discrete sub-compartments have been recently visualized in *T. gondii* through a family of proteins named IMC sub-compartment proteins (ISPs) located either at the apical cap, in the middle part of the IMC or in a basal region lying in the last third of the parasite length [5]. In addition, Coccidians possess a conoid, a motile organelle composed of tubulin fibers arranged in spiral at the apical pole [6], [7]. At the opposite pole, the basal complex remains more enigmatic but is composed of a basal polar ring where the membrane occupation and recognition nexus protein 1 (MORN1) localizes and a posterior cup where centrin 2 is found [8]. In *Toxoplasma*, both apical and basal complexes originate close to the centrosomes very early in the cell division process, and during the development of the daughter cells their basal complex appears as a ring structure that migrates to the basal pole and constricts in the mature parasite [8].

Most invasive apicomplexan zoites exhibit a unique substrate-dependent motion referred to as gliding motility, which allows parasites to cross non-permissive biological barriers and assists host cell invasion and egress from infected cells. The force generated by the parasite to propel itself inside a target cell originates from a conserved actomyosin machinery termed the glideosome that is located at the pellicle, in the limited space between the PM and the IMC [9]. The glideosome sustains the forward movement of the parasite by rearward translocation of adhesins that are apically secreted by the micronemes and bound to host cell receptors [10]. In *Toxoplasma*, the molecular motor complex is composed of the myosin heavy chain A (MyoA) and two associated light chains, the myosin light chain 1 (MLC1) and the essential light chain 1 (ELC1) [11]–[13]. This complex is recruited to the IMC via association with the C-terminal domain of the gliding-associated protein GAP45 [14]. While two integral membrane proteins of the IMC, GAP40 and GAP50, secure a firm anchoring of the complex in the outer membrane of the IMC, the N-terminal domain of GAP45 is fluidly inserted into the PM through two lipid modifications, myristoylation and palmitoylation [14]–[16]. The central sequence of GAP45 adopts an extended coiled-coil conformation that critically maintains the cohesion between the PM and the IMC during glideosome function, holding the two membranes at an optimal and constant distance [14]. Recently, *MyoA* was excised by DiCre recombinase in *T. gondii* and clones have been obtained establishing that this motor is dispensable for parasite survival, however their invasion rate was reduced by 80% [17]. In contrast *MyoA* could not be permanently excised in a parasite mutant lacking the gene coding for the two alternative spliced variants of the class XIV myosin B (MyoB) and myosin C (MyoC). Moreover the genes coding for actin (ACT1), MLC1 and GAP45 were conditionally excised but similarly, the parasites failed to be cloned, indicative of their essentiality [18].

GAP70 is a protein closely related to GAP45, which is found only in Coccidians and localizes exclusively to the apical cap [14]. Like GAP45, GAP70 is anchored by its N-terminal acylation to the PM and by its C-terminus to the apical IMC. It recruits MyoA but exhibits a longer coiled-coil domain and only partially complements GAP45 inducible knockout (GAP45-iKO) [14]. GAP70 is presumably tailored to accommodate a longer distance between the PM and the IMC. While GAP45 is essential for the lytic cycle of the parasite, GAP70 can be deleted without noticeable phenotype, likely due to a compensatory effect of the abundant GAP45 [14]. The C-terminal domains of GAP45 and GAP70 are very similar, and hence the specific determinant that targets GAP70 to the restricted area of the IMC remains unknown.

Coccidians possess a third member of this family, GAP80, which is shown here to localize to the posterior pole of *T. gondii* tachyzoites. GAP80 assembles a new glideosome around MyoC (MyoC-glideosome). Characterization of the partners interacting with GAP80 led to the identification of IMC-associated protein 1 (IAP1), a key determinant for the assembly of the MyoC-glideosome at the posterior polar ring. While this complex is dispensable for parasite survival, disruption of its individual components was strikingly compensated by the assembly of a chimeric glideosome composed of components of the MyoA- and MyoC-glideosomes. These findings shed light on the complexity and versatility of the gliding machine in the coccidian subgroup of Apicomplexa.

## Results

### GAP80 belongs to a small family of GAP45 related proteins

*TgGAP70* (TGME49_233030) [14] and *TgGAP80* (TGME49_246940) code for proteins showing considerable sequence similarity with GAP45 but which are restricted to the coccidian *Toxoplasma*, *Neospora* and *Sarcocystis*, in contrast to *GAP45* which is found across the whole Apicomplexa phylum. The amino acid sequence alignment of these family members highlighted a significant conservation in the extreme C-terminus, which has been implicated in the interaction between GAP45 and MLC1-MyoA [14] (Figure S1A). In contrast to GAP45 and GAP70, the central region of GAP80 is not predicted to adopt a coiled-coil conformation probably due to the high content of proline residues (14% versus less than 3% in GAP45 and GAP70) and is instead predicted to fold into several short alpha helices (Figure S1B and file S1).

**Figure 1:**
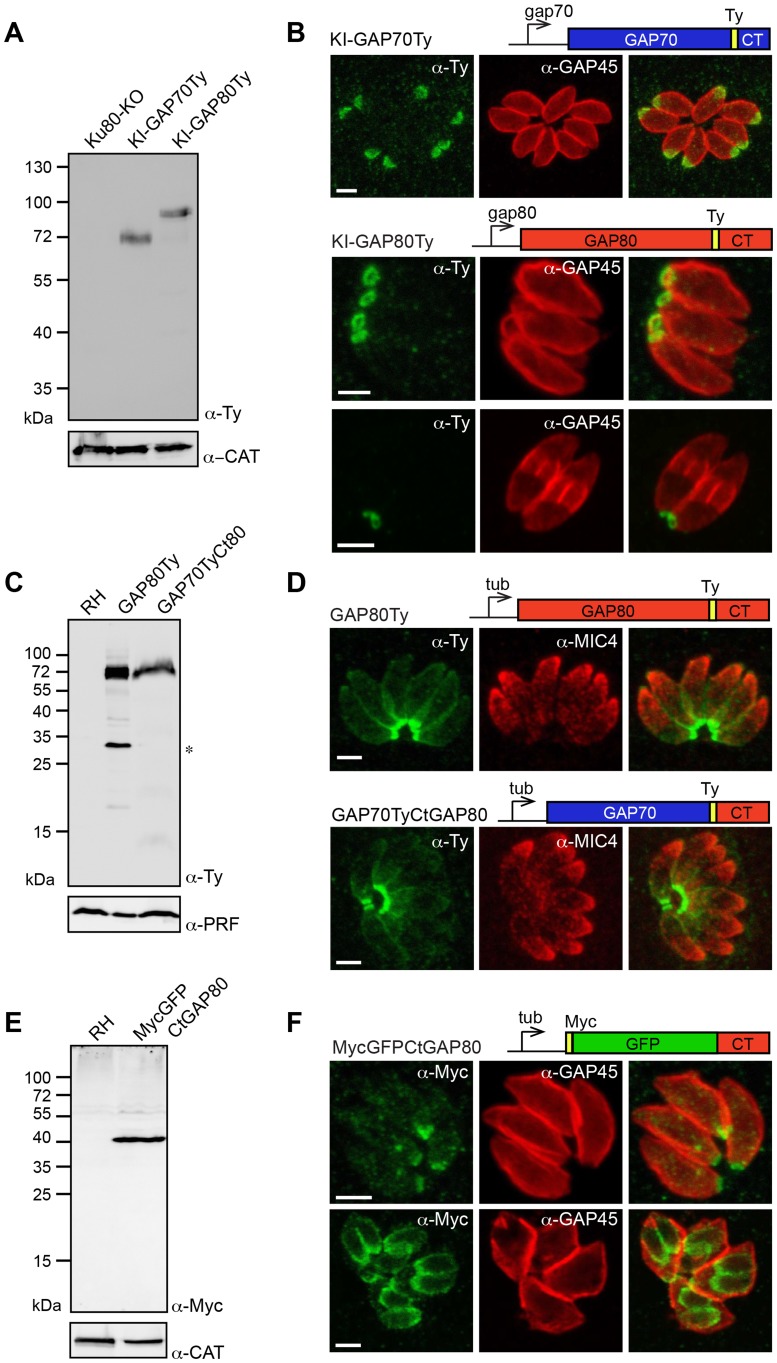
A family of GAPs anchored in different sub-compartments of the IMC. A. Total lysates from Ku80-KO parasites expressing a Ty-tagged endogenous *GAP70* or *GAP80* (KI: knock-in) analyzed by western blot using anti-Ty antibodies and catalase (CAT) as loading control. B. Localization of KI-GAP70 at the apical cap and KI-GAP80 in a ring-like structure at the basal pole assessed in intracellular parasites using anti-Ty as well as anti-GAP45 or anti-IMC1 antibodies that stain the periphery or the IMC, respectively. Scale bars: 2 µm. C. Immuno-blot of total lysates of RH parasites expressing a second copy of GAP80Ty or GAP70TyCtGAP80 under the control of the tubulin (Tub) promoter. Profilin (PRF) was used as a loading control. The star indicates a degradation product. D. Localization of the second copy of GAP80Ty or GAP70TyCtGAP80 assessed in intracellular parasites using anti-Ty together with anti-MIC4 antibodies that stain the micronemes. Scale bars: 2 µm. E. Total lysates from RH parasites expressing MycGFPCtGAP80 under the control of the tubulin promoter analyzed by western blot using anti-Myc antibodies and CAT as a loading control. F. Localization of MycGFPCtGAP80 at the posterior sub-compartment of the IMC in mature parasites and in growing daughter cells. Scale bars: 2 µm.

A knock-in (KI) strategy in Ku80-KO recipient strain [19], [20] was designed to insert a Ty-tag just upstream of the conserved C-terminal region of GAP70 (KI-GAP70Ty) and GAP80 (KI-GAP80Ty), respectively (Figure S1C). Stable parasite lines confirmed that both genes are expressed in the tachyzoite stage (Figure 1A). GAP80 exhibited the same abnormal migration behavior on SDS-PAGE as previously reported for GAP45 and GAP70 with an apparent molecular weight of 80 kDa whereas the predicted size is 45 kDa. Epitope tagging of GAP70 at the endogenous locus confirmed localization to the apical cap of the parasite previously reported based on expression of a second epitope-tagged copy [14]. In sharp contrast, GAP80 localized exclusively to the basal pole of mature parasites and showed a ring-shaped staining corresponding to the posterior polar ring (Figure 1B). To determine if the C-terminus of GAP80 was sufficient to confer the posterior localization, this domain consisting of the last 85 amino acids (aa) of the protein was either fused to GFP (MycGFPCtGAP80) or exchanged with the corresponding C-terminal domain of GAP70 (GAP70TyCtGAP80) and expressed as a second copy (Figure 1 C, E). As a control, expression of a second copy of GAP80Ty was found mainly targeted to the basal pole, opposite to the apical microneme staining of MIC4, and also slightly at the parasite periphery due to overexpression (Figure 1D). Exchange of the C-terminal domain in GAP70TyCtGAP80 conferred a posterior localization to the otherwise apically localized GAP70 (Figure 1D). MycGFPCtGAP80 also targeted to the basal polar ring, confirming that this C-terminal domain was sufficient to act as a targeting determinant (Figure 1F). As previously observed for the C-terminus of GAP45, the C-terminal domain of GAP80 alone was detectable in the nascent IMC of the daughter cells whereas the full-length proteins were found in the mature pellicle only (Figure 1F). This restriction is likely due to the absence of N-terminal acylation (bioinformatically predicted) in the case of MycGFPCtGAP80, which would anchor GAP80 to the PM prior to its association with the basal pole [14].

### GAP80 belongs to the MyoC-glideosome

**Figure 2:**
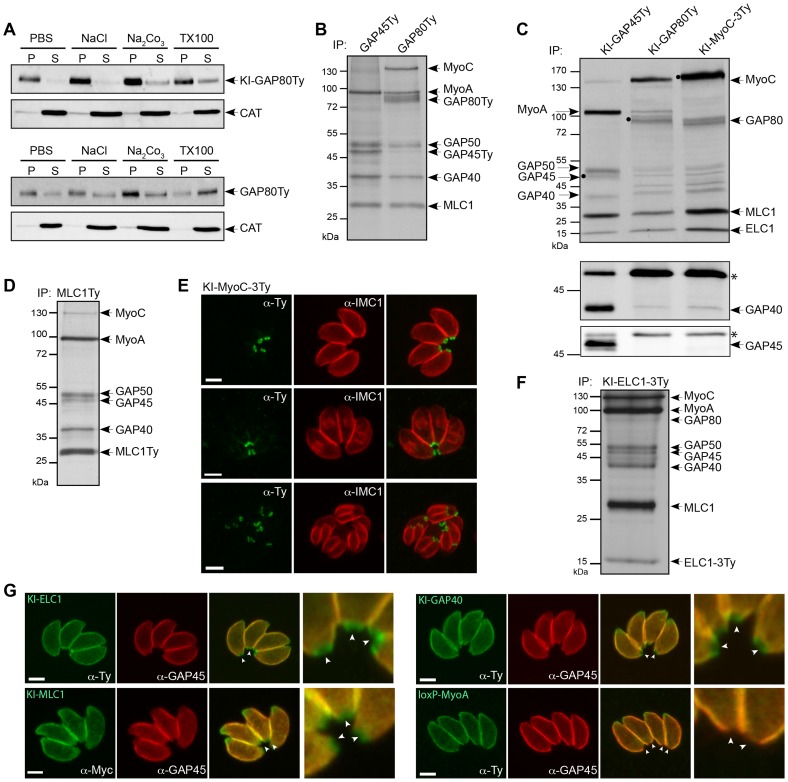
Two myosin light chains shared between two motors. A. The solubility of GAP80 constructs (KI-GAP80Ty and GAP80Ty) was assessed by fractionation after extraction in PBS, PBS/NaCl, PBS/Na_2_CO_3_ or PBS/Triton X-100. Their distribution in different fractions was assessed by western blot using anti-Ty antibodies and the soluble catalase (CAT) as control for the correct fractionation. B. Parasites stably expressing a second copy of GAP45Ty and GAP80Ty were labeled with [^35^S]-methionine/cysteine and subjected to co-IP with anti-Ty antibodies. Eluted proteins were visualized by autoradiography. A protein above 130 kDa was additionally found in GAP80Ty elution and identified as MyoC. C. Metabolically labeled parasites endogenously Ty-tagged at the *GAP45*, *GAP80* or *MyoC* locus have been subjected to co-IP with anti-Ty antibodies. The black circles correspond to the respective bait. The same elution fractions were analyzed by western blot using anti-GAP40 and anti-GAP45 antibodies, respectively. The asterisks indicate the Ig heavy chains that cross-react with the antibodies. D. Autoradiography of labeled parasites stably expressing a second copy of MLC1Ty after co-IP experiment with anti-Ty antibodies. E. Localization of the endogenous MyoC in a ring-like structure at the basal pole of mature parasites and late stage daughter cells using anti-Ty and anti-IMC1 antibodies. Scale bars: 2 µm. F. Autoradiography of labeled parasites stably expressing a Ty-tagged version of the endogenous ELC1 (KI-ELC1-3Ty) after co-IP performed with anti-Ty antibodies. Scale bars: 2 µm. G. Localization of the endogenously tagged ELC1, MLC1 and GAP40 and of loxP-TyMyoA assessed in intracellular parasites using anti-Ty and anti-GAP45 antibodies. Arrowheads point to the basal end of the parasites that are also presented in the magnifications.

To ascertain the association of GAP80 with the membrane, fractionation experiments were completed. While KI-GAP80Ty was insoluble in PBS and high salt, it was partially solubilized in carbonate indicative of a peripheral protein that can also be partially extracted in the non-ionic detergent Triton X-100 (Figure 2A). GAP80Ty expressed as a second copy was more readily extracted in the various conditions, likely due to a looser IMC interaction caused by the overexpressed fraction that localizes to the periphery of the parasite and lacks basal-specific anchor(s).

To identify the interacting partners of GAP80, co-immunoprecipitation (co-IP) experiments were performed in the presence of Triton X-100 using anti-Ty antibodies on ^35^S-methionine and -cysteine metabolically labeled parasites expressing a second copy of either GAP80Ty or GAP45Ty as control. The eluted fractions showed similar profiles for MLC1, MyoA, GAP40 and GAP50 but an additional protein migrating around 130 kDa appeared only in the GAP80Ty co-IP (Figure 2B). The same profiles were also obtained for the co-IPs performed with the KI-GAP80Ty and KI-GAP45Ty strains [21] (Figure 2C) and the presence of MLC1, MyoA and GAP40 were verified by western blot analyses (Figure S2A). However, in the KI-GAP80Ty elution, GAP40 is much less abundant than within the MyoA-glideosome and the absence of GAP45 was confirmed by immunoblot despite the presence of a visible band at a similar size on the autoradiograph (Figures 2C). To validate that most of the glideosome components were shared between the two complexes, a reverse co-IP experiment was carried out on metabolically labeled parasites expressing a second tagged copy of the shared MLC1 (MLC1Ty). All the components of the glideosome were again present in the bound fraction including the protein migrating at around 130 kDa (Figure 2D). Mass spectrometry analyses confirmed that the band around 80 kDa corresponded to GAP80 and identified the protein migrating above 130 kDa (Figure S2B) to be encoded by TGME49_255190 (ToxoDB, [22]). This gene, previously described as the myosin B/C (*MyoB/C*), gives rise to two alternatively spliced products, MyoB and MyoC that differ in the length of their tail domain and in their localization [23]. MyoB/C was identified from 31 unique peptides covering 33% of the sequence (Table S1) and including one peptide (DVSYLIGMLFQR) specific to MyoC that was previously reported to be the predominant product expressed in the tachyzoite stage [23]. MyoC was C-terminally tagged in the endogenous locus and as previously observed with the expression of a second tagged-copy [23], MyoC was visible to the posterior polar ring of mature parasites and in the “late” stage developed daughter cells once the most basal sub-compartment of the IMC is built (Figure 2E). In contrast to GAP80-Ty, which is able to associate with a limited amount of MyoA, co-IPs performed with KI-MyoC-3Ty established that MyoC only interacts with GAP80, likely GAP50, ELC1 and MLC1 whereas GAP45 and MyoA are absent (Figure 2C). Since the GAPs are migrating in close proximity, western blot analyses on the co-IP materials were performed to confirm the presence of GAP40 and the absence of GAP45 in the MyoC complex. The association of MyoC with GAP80Ty was further confirmed by western blot analysis of the co-IP, using anti-MyoC antibodies (Figure S2C).

To determine if ELC1 was interacting with MyoC in addition to MyoA, a strain in which the endogenous gene was tagged at its C-terminus by knock-in was generated (KI-ELC1-3Ty) and the co-IP experiment performed using anti-Ty antibodies immunoprecipitated the entire glideosome including GAP80 and MyoC (Figure 2F). Finally, localization of endogenous ELC1, MLC1 and GAP40 clearly showed a staining posterior to that of GAP45 and corresponding to the location of the MyoC-glideosome (Figures 2G and S2D). In contrast, the signal for MyoA perfectly co-localized with GAP45 and was absent from the basal end (Figure 2G).

Taken together these findings identified a new coccidian-specific glideosome named the MyoC-glideosome, which shares the anchoring components to the IMC with the MyoA-glideosome, broadly conserved across the phylum of Apicomplexa. While GAP70 is part of the MyoA-glideosome at the apical cap [14], GAP80 belongs to the MyoC complex located at the posterior polar ring. MyoC belongs to the unconventional class XIV and appear to share the same myosin light chains, MLC1 and ELC1, with MyoA.

### *T. gondii* IAP1 targets the MyoC-glideosome to the basal polar ring

Given the largely shared composition of the MyoC-glideosome with the MyoA-glideosome, we reasoned that either GAP80 and/or a yet unidentified component should act as trafficking determinant(s) to confine the MyoC-glideosome to the posterior polar ring.

**Figure 3:**
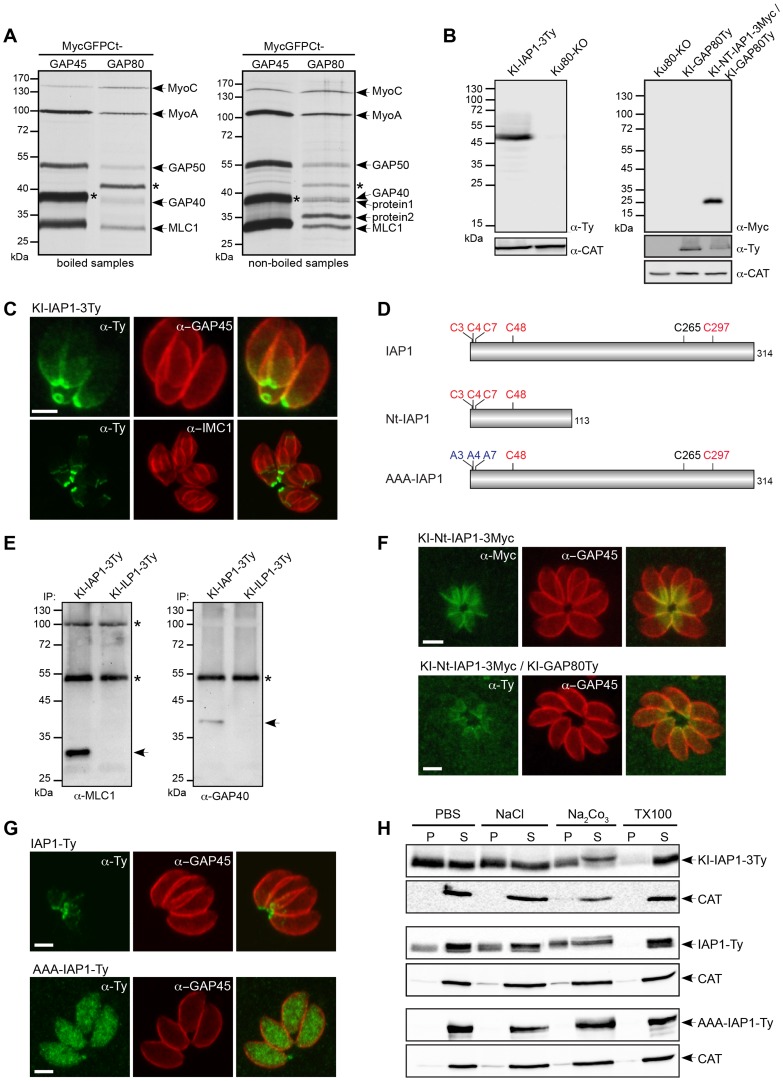
Identification of an IMC-associated protein recruiting the MyoC-glideosome to the basal polar ring. A. Parasites stably expressing MycGFPCtGAP45 and MycGFPCtGAP80 as a second copy were subjected to co-IP with anti-Myc antibodies after metabolic labeling. Eluted proteins were boiled (left panel) or not (right panel) before migration on a SDS-page gel and then visualized by autoradiography. Stars indicate the Myc-tagged proteins. Proteins 1 and 2 are the candidates analyzed by mass spectrometry. B. Total lysates from parasites expressing an endogenous Ty-tagged full-length version of IAP1 (KI-IAP1-3Ty) in a Ku80-KO background or an endogenous Myc-tagged truncated version of IAP1 (KI-Nt-IAP1-3Myc) in the KI-GAP80Ty were analyzed by western blot using respectively anti-Ty and anti-Myc antibodies and the catalase as loading control. C. Localization of KI-IAP1-3Ty at the posterior polar ring and in the last third of the IMC assessed in intracellular parasites using anti-Ty, anti-GAP45 and anti-IMC1 antibodies. Scale bar: 2 µm. D. Schematic representation of the IAP1 constructs used in this study and highlighting the position of the cysteine residues. In red are the cysteines predicted to be palmitoylated by CSS-Palm 3.0 with the highest threshold [26] and in blue are the ones mutated to alanines. E. Co-IP performed with anti-Ty antibodies on parasites expressing KI-IAP1-3Ty and KI-ILP1-3Ty (as control) and revealed by western blot using anti-MLC1 and anti-GAP40 antibodies. Arrows show MLC1 and GAP40, respectively. The asterisks indicate the Ig heavy and light chains that cross-react with the antibodies. F. The truncated version of IAP1 (KI-Nt-IAP1-3Myc) was generated in wild type and KI-GAP80Ty backgrounds. It localizes in the last third of the IMC and led to the relocalization of GAP80Ty in the same area of the IMC. Scale bars: 2 µm. G. A wild type second copy of IAP1 (IAP1-Ty) stably expressed in RH strain localized to the posterior part of the IMC while a second copy of IAP1 mutated on the three first cysteines (AAA-IAP1-Ty) was found in the cytoplasm. Scale bars: 2 µm. H. The solubility of strains expressing IAP1 (KI-IAP1-3Ty, IAP1-Ty and AAA-IAP1-Ty) was assessed by fractionation after extraction in PBS, PBS/NaCl, PBS/Na_2_CO_3_ or PBS/Triton X-100. Their distribution in different fractions was assessed by western blot using anti-Ty antibodies and the soluble catalase (CAT) was used as control for the correct fractionation.

The C-terminal domain of GAP80 was sufficient to target GFP to the posterior pole and likely also sufficient to recruit the MyoC complex, as it is the case for the MyoA complex with GAP45. Toward the identification of a specific component anchoring the MyoC-glideosome to the basal sub-compartment of the IMC, we completed co-IP experiments with anti-Myc antibodies on parasite strains expressing either MycGFPCtGAP80 or the control MycGFPCtGAP45 (Figure 3A). MycGFPCtGAP80 efficiently immunoprecipitated MyoC, MyoA, MLC1, GAP40 and GAP50. Importantly, two additional components associated with MycGFPCtGAP80 became clearly visible when the samples were not boiled prior to loading on SDS-PAGE suggesting proteins with TMD or strongly associated with membranes [24], [25]. Preparative co-IPs were then performed with MycGFPCtGAP80 and MycGFPCtGAP70 as a control in order to identify the putative anchorage(s) (Figure S3A). Two bands, one below 40 kDa (protein 1) and one above 35 kDa (protein 2), were cut out and 8 and 9 proteins were identified by mass spectrometry, respectively (Table S2). Besides obvious contaminants corresponding to the abundant surface protein SAG1, heat shock and ribosomal proteins, peptides corresponding to MLC1 and GAP50 were also found. More interestingly, peptides corresponding to three hypothetical genes present only in Coccidians and exhibiting a similar cell cycle transcription profile as MyoC were identified and investigated further by epitope tag knock-in at the endogenous locus. The TGME49_283510 product was the only candidate localized to the posterior polar ring and the basal sub-compartment of the IMC and was named IAP1 for IMC-associated protein 1 (Figure 3 B, C and table S3). No transmembrane spanning domain was apparent for IAP1 but instead five cysteine residues were predicted to be palmitoylated with a high probability [26], supporting the strong interaction with the IMC (Figures 3D and S3B). In addition, acylation at multiple sites could explain why IAP1 migrated higher than its expected size, a shift that was even more pronounced when one (Figure S3C) or three acidic Ty-tags (Figure 3B) were added.

To demonstrate that IAP1 belongs to the MyoC-glideosome, parasites expressing KI-IAP1-3Ty were used to perform a co-IP together with the IMC-localized protein ILP1 [27] also tagged similarly at the endogenous locus (KI-ILP1-3Ty) and used as a negative control (Figure S3D). Western blot analyses revealed the presence of MLC1 and GAP40 in the bound fraction of KI-IAP1-3Ty but not in the control KI-ILP1-3Ty strain (Figure 3E). The presence of MyoC in this complex was visualized by autoradiography of a co-IP performed on metabolically labeled parasites expressing KI-IAP1-3Ty (Figure S3E).

To unravel how IAP1 associates with the IMC, we examined the contribution of four out of the five predicted palmitoylated cysteine residues lying in the N-terminal part of the protein (Figure 3D). A truncated version of IAP1 encompassing the 113 first residues (KI-Nt-IAP1-3Myc) was generated by knock-in in the Ku80-KO as well as in the KI-GAP80Ty background (Figure 3B, D). KI-Nt-IAP1-3Myc was still anchored to the posterior pole of the parasite but lost the polar ring localization and concomitantly GAP80 relocalized from the polar ring to the broader basal sub-compartment of the IMC (Figure 3F). To more directly analyze the contribution of the N-terminal cysteine residues in IAP1 anchoring, a second copy of IAP1 mutant exhibiting C3, C4 and C7 changed to alanine residues (AAA-IAP1-Ty, Figure 3D), controlled by tubulin promoter, was stably expressed. In contrast to its wild type counterpart that localized to the basal polar ring, AAA-IAP1-Ty was found in the cytoplasm (Figure 3G). In addition, this mutant was completely soluble in PBS while the wild type protein (endogenous or second copy) was fully extracted only in the presence of detergent (Figure 3H). Taken together, these data established that IAP1 is a component of the MyoC-glideosome that contributes to its basal polar ring localization most likely via N-terminal palmitoylation.

### Substitution mechanisms compensate for the deletion of MyoC-glideosome components

To gain insight into the function of the MyoC-glideosome without impacting on the MyoA-glideosome, *MyoC*, *GAP80* and *IAP1* were targeted for genetic disruption.

**Figure 4:**
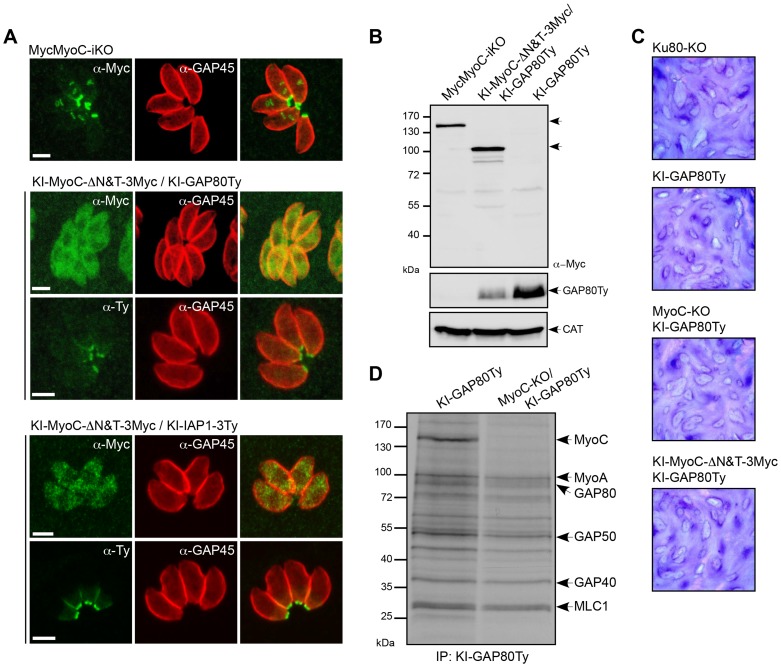
MyoC is dispensable in tachyzoites. A. Localization of endogenous MyoC N-terminally Myc-tagged (MycMyoC-iKO) and of endogenous truncated MyoC (KI-MyoC-ΔN&T-3Myc) expressed in KI-GAP80Ty or KI-IAP1-3Ty. Scale bars: 2 µm. B. Total lysates from parasites expressing MycMyoC-iKO and KI-MyoC-ΔN&T-3Myc/KI-GAP80Ty were analyzed by western blot with anti-Myc antibodies. Parasites expressing KI-GAP80Ty were loaded as a control and CAT as a loading control. C. Plaque assays performed with Ku80-KO, KI-GAP80Ty, MyoC-ΔN&T-3Myc/KI-GAP80Ty, MyoC-KO/KI-GAP80Ty cell lines and fixed after 7 days. No defect in the lytic cycle was observed. D. Co-IP experiments performed with anti-Ty antibodies on cell lines metabolically labeled and expressing KI-GAP80Ty in a wild type and in a MyoC-KO background.

We first generated an N-terminal tagged version of the full-length MyoC by replacing the endogenous promoter by a Tet-inducible one in the TATi strain (MyoC-iKO) and a truncated version lacking the neck and tail domains (KI-MyoC-ΔN&T-Myc) by single homologous recombination in the *MyoC* locus (Figure S4A, B). In contrast to wild type MyoC that localized to the posterior polar ring of mature parasites and growing daughter cells, MyoC-ΔN&T-Myc was cytosolic (Figure 4A). Deletion of the neck and tail domains of MyoC destabilized GAP80 as shown by the reduced amount of GAP80 detectable by western blot (Figure 4B) but did not impact on GAP80 or IAP1 basal localization (Figure 4A). In addition, no noticeable phenotype has been observed during the lytic cycle by plaque assay (Figure 4C). Given the previous association of MyoB/C with pellicle integrity during cell division [23], we examined the rate of replication by counting the number of parasites per vacuole 24 hours post invasion (Figure S5A). This mutant showed no defect in intracellular growth and no impairment in egress (Figure S5B).

Since MyoC-ΔN&T-Myc showed no loss of fitness, a conventional knockout was produced (MyoC-KO) by double homologous recombination (Figure S5 C, D). Given the position of MyoC at the basal polar ring, we monitored by time-lapse microscopy the ability of MyoC-KO parasites to perform twirling during an induced egress assay and observed no defect compared to the wild type strain (Videos S1 and S2). We finally compared the co-IP of KI-GAP80Ty from a wild type and a MyoC-KO strain and confirmed the absence of MyoC while the rest of the complex was still assembled (Figure 4D). Importantly, the interaction of MyoA with GAP80 suggested that this motor had the potential to substitute for the absence of MyoC at the posterior polar ring. Since GAP80 level is lower in the absence of MyoC, it was not possible to make a quantitative comparison of the co-IPs between the two parasite lines.

**Figure 5:**
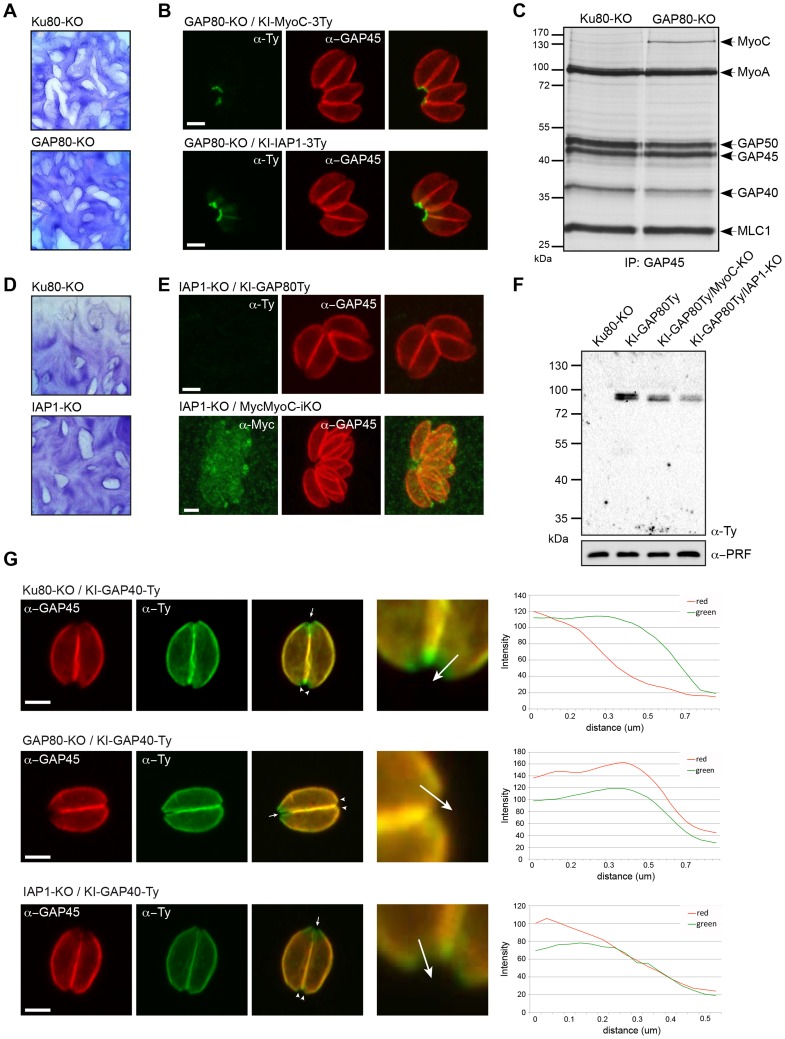
The MyoC-glideosome is dispensable in tachyzoites. A. No growth defect was detected 7 days post-invasion by plaque assays performed with Ku80-KO and GAP80-KO strains. B. In absence of GAP80, endogenous MyoC and IAP1 (KI-MyoC-3Ty and KI-IAP1-3Ty) are still localized to the basal polar ring. Scale bars: 2 µm. C. Co-IP experiments performed with anti-GAP45 antibodies on Ku80-KO and GAP80-KO strains after metabolic labeling with [^35^S]-methionine/cysteine. D. Plaque assays performed with Ku80-KO and IAP1-KO cell lines and fixed after 7 days. E. Immunofluorescence assays performed on intracellular parasites showing that in absence of IAP1, MyoC and GAP80 (MycMyoC-iKO and KI-GAP80Ty) are not localized to the basal polar ring anymore. Scale bars: 2 µm. F. Western blot analysis of total extract of parasites expressing KI-GAP80Ty in 3 different backgrounds. G. Localization of GAP45 in Ku80-KO, GAP80-KO and IAP1-KO cell lines relatively to GAP40Ty showing that in absence of IAP1 or GAP80, GAP45 staining goes further down to the basal complex as illustrated by the magnifications of the posterior poles and the RGB profile plots determined using ImageJ along the arrow. Scale bars: 2 µm. Little arrows point to the apical pole of the parasites while arrowheads point to the posterior pole.

To gain further information about the MyoC-glideosome, a conventional knockout of the *GAP80* gene was generated in the Ku80-KO strain (Figure S5E, F). The absence of phenotype by plaque assay indicated that GAP80-KO parasites were able to accomplish their lytic cycle normally (Figure 5A) and indeed the individual steps including intracellular growth and egress were not altered (Figure S5 G, H). Surprisingly, upon deletion of *GAP80*, neither MyoC nor IAP1 showed an altered localization (Figure 5B). Given the homology between GAP80 and GAP45, it appeared plausible that GAP45 could compensate for the deletion of GAP80. We tested this hypothesis by performing a co-IP using anti-GAP45 antibodies on metabolically labeled wild type parasites and GAP80-KO (Figure 5C). In addition to the MyoA-glideosome components precipitated in the Ku80-KO strain, MyoC was precipitated in GAP80-KO parasites only, confirming that in this mutant strain GAP45 was able to interact with MyoC and hence possibly compensates for the absence of GAP80.

Finally, we generated an IAP1-KO strain in the Ku80-KO background (Figure S6A, B) as well as in MyoC-iKO and KI-GAP80Ty backgrounds. Since IAP1 is involved in the recruitment of the MyoC-glideosome to the basal polar ring, it has no known counterpart in the MyoA-glideosome to rescue its deletion. No loss of parasite fitness was monitored in the absence of IAP1 (Figure 5D) or in intracellular growth and egress (Figure S6C, D). However, in the absence of IAP1, GAP80 was no longer detectable at the basal polar ring or elsewhere in the parasite (Figure 5E) but remained detectable by western blot even though it appeared less abundant, likely due to its reduced stability in the absence of the complex (Figure 5F). MyoC was also absent from the basal polar ring and instead localized to the cytoplasm, at the periphery and also concentrated at the apical polar ring (Figure 5E). Strikingly, GAP45, a component of MyoA-glideosome clearly extended its localization to the basal end of GAP80-KO and IAP1-KO mutants as shown by the labeling of GAP45 in the posterior area, which is normally excluded in the Ku80-KO background strain (Figure 5G).

Individual deletion of the components of the MyoC-glideosome for which counterparts exist in the MyoA-glideosome are compensated for by the formation of a chimeric glideosome attesting to the adaptability and versatility of *T. gondii*. Moreover, deletion of IAP1, the protein responsible for the recruitment of GAP80-MyoC-MLC1-ELC1 to the basal polar ring, leads to the relocalization of at least some components of MyoA-glideosome to possibly compensate for the absence of a motor at the basal pole.

### A non-functional MyoC fails to incorporate into the basal glideosome complex

**Figure 6:**
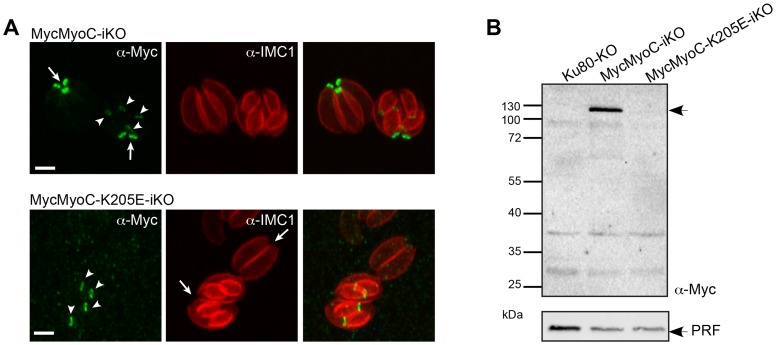
A non-functional MyoC fails to incorporate into the basal glideosome complex. A. Localization of MycMyoC-iKO and MycMyoC-K205E-iKO in dividing parasites stained with an IMC marker shows that the mutated MyoC is not incorporated in the mature parasites. Scale bars: 2 µm. B. Western blot analysis of total extract of intracellular parasites expressing MycMyoC-iKO and MycMyoC-K205E-iKO. The mutated MyoC is never detected with anti-Myc antibodies.

To tackle MyoC function, it was necessary to hamper MyoA incorporation into the basal glideosome to avoid a compensatory effect. To achieve that, we thought of introducing a non-functional point mutation in the ATP-binding site of endogenous MyoC. This parasite line was created using the same double homologous strategy as for the MyoC-iKO strain except that in the N-terminal homology fragment previously used to recombine, the ATP-binding site GESGAGKT was mutated to GESGAGET (Figure S4B, C). This mutation in the P-loop of MyoC (MyoC-K205E) is predicted to interfere with ATP-binding and thus should result in strong actin binding in combination with a lack of motile activity. To ensure the integration of the mutation, the N-terminal MyoC fragment was synthetized with a different codon usage up to and including the mutated ATP-binding site and the homologous region was lying downstream (Figure S4C). Additionally, to enhance the efficiency of the recombination, the locus was targeted with a specific guide RNA (gRNA) CRISPR-CAS9 plasmid [28]. Stable MycMyoC-K205E parasites were obtained (Figure S4D) and two clones from independent transfections were sequenced for the presence of the mutation. Surprisingly MyoC-K205E failed to localize to the basal polar ring of mature parasites as previously described [23] (Figure 6A). Instead, MyoC-K205E was clearly visible in all the late stage developed daughter cells identified with IMC1 (Figure 6A) implying that the protein is expressed and targeted to the basal polar ring during division but is not incorporated in this structure in the mature parasites. MyoC-K205E is not detectable by western blot (Figure 6B) suggesting that it is destabilized when not incorporated into the basal pole of mature parasites. Parasites expressing a non-functional MyoC have no phenotype in intracellular growth, invasion or egress (Figure S4 E-G). These data suggest that functional MyoC might be necessary for its integration in the basal pole. The expression of a non-functional MyoC led to a situation similar to the deletion of MyoC, leaving again physical room for a compensatory mechanism.

### GAP80 can substitute for GAP45 in MyoA-glideosome function

**Figure 7:**
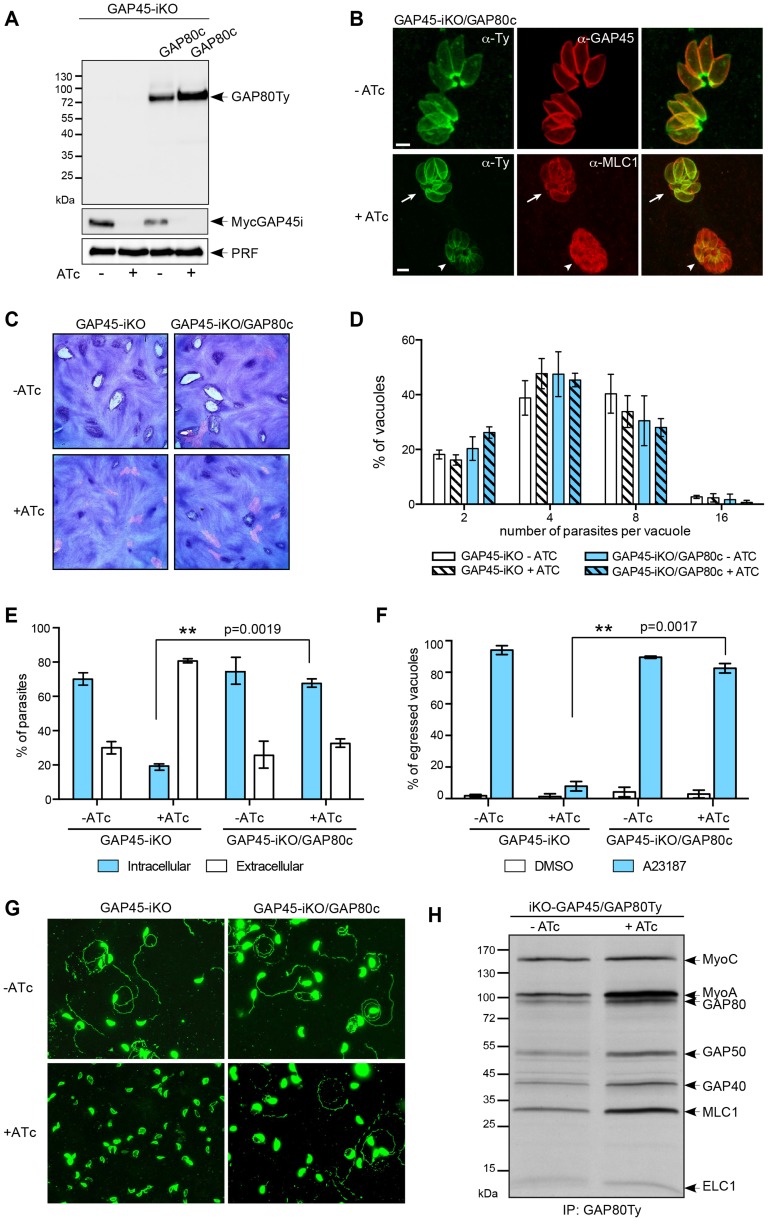
GAP80 is able to recruit MLC1-MyoA and to preserve the cohesion of the pellicle. A. Western blot analysis showing the regulation of the inducible GAP45 (GAP45i), the expression of the complementing Ty-tagged copy of *GAP80* (GAP80c) expressed under a tubulin promoter and its stabilization upon addition of ATc for 48 hours. B. Immunofluorescence assay performed on GAP45-iKO/GAP80c strain in absence and in presence of ATc. The arrow indicates a vacuole in which GAP80Ty complements GAP45 deletion and recruits MLC1 at the pellicle while the arrowhead points out a vacuole in which the level of expression of GAP80Ty is not sufficient to rescue GAP45 depletion. Scale bars: 2 µm. C. Plaque assays performed with GAP45-iKO and GAP45-iKO/GAP80c strains treated ± ATc for 7 days. GAP80 restores partially the growth defect of GAP45 depletion. D. Intracellular growth assay performed on GAP45-iKO and GAP45-iKO/GAP80c strains by determining the number of parasites per vacuole after 48 hours ± ATc. Data are represented as mean ± SD. E. Invasion capacity of GAP45-iKO and GAP45-iKO/GAP80c strains was evaluated using a two-color immunofluorescence assay performed after 42 hours ± ATc. Intracellular: invaded parasites, extracellular: attached parasites. Data are represented as mean ± SD. F. Ionophore-induced egress assay of GAP45-iKO and GAP45-iKO/GAP80c strains performed by treating the parasites with DMSO or A23187 for 5 min after 56 hours ± ATc. Results are expressed as a percentage of ruptured vacuoles and represented as mean ± SD. For E and F, the significance of the results was assessed using a parametric paired t-test and the two-tailed p-values are written on the graphs. G. Gliding assay performed with GAP45-iKO and GAP45-iKO/GAP80c strains after 42 hours ± ATc. H. Co-IP carried out with anti-Ty antibodies on GAP45-iKO/GAP80c strain metabolically labeled after 48 hours ± ATc. Upon GAP45 depletion, GAP80 is integrated in MyoA-glideosome and recruits MyoA while the level of bound MyoC remains unchanged.

Given the overall similarities in the architecture and composition of the two glideosomes, it appeared legitimate to assume that the MyoC-glideosome participates in some aspects of the gliding function possibly exemplified by the stationary twirling where the parasite rotates, contacting the substrate via its posterior pole [29]. To support this notion we first anticipated that GAP80 could functionally complement the depletion of GAP45 when expressed at a suitable level. A second copy of GAP80 (GAP80Ty) was therefore introduced in the inducible knockout of GAP45 (GAP45-iKO) [14] and expressed under the control of the tubulin promoter (Figure 7A). Overexpression of GAP80Ty led to an overflow of the protein to the entire pellicle in addition to the basal polar ring and to a rescue of the recruitment of MLC1-MyoA to the pellicle upon depletion of MycGAP45i in the presence of anhydrotetracycline (ATc) (Figure 7B). This correlated with the partial complementation seen by plaque assay (Figure 7C) and the normal intracellular growth curve (Figure 7D) [14]. Moreover, GAP80Ty was able to complement both the invasion and egress defects caused by GAP45 deletion to levels almost comparable to the controls (Figure 7E, F and Table 1). The ability of GAP80 to restore the motility defect linked to depletion of GAP45 upon ATc treatment was assessed in the gliding trail assay using anti-SAG1 antibodies to detect the trails (Figure 7G). GAP80Ty is predominantly associated with the MyoA-glideosome upon GAP45i depletion while the level of assembly with MyoC remains constant (Figure 7H).

**Table 1:** Summary of the phenotypes observed in this study.

Strain of interest	Intracellular growth	Egress	Invasion	Gliding 2
**GAP45-iKO -ATc** 1	Normal	88.9±7.2	73.0±4.2	+
**GAP45-iKO +ATc** 1	Normal	7.3±0.8	20.1±1.1	-
**GAP45-iKO/GAP80c -ATc**	Normal	89.5±0.7	74.3±8.5	+
**GAP45-iKO/GAP80c +ATc**	Normal	82.5±3.0	67.5±2.6	+
**GAP45-iKO/GAP80-KO -ATc**	Normal	84.3±2.1	80.3±6.0	+
**GAP45-iKO/GAP80-KO +ATc**	Normal	5.5±2.7	9.7±0.3	-
**MyoC-iKO -ATc**	Normal	90.0±5.7	77.0±4.2	+
**MyoC-iKO +ATc**	Normal	85.0±2.8	82±2.8	+
**MyoC-iKO/MyoA-KO -ATc**	Normal	7.5±5.7	9.8±1.8	-
**MyoC-iKO/MyoA-KO +ATc**	Normal	5.0±0	1.8±0.4	-

1 For GAP45-iKO, values are the mean of the assays presented in figures 5 and 6;

2 Gliding refers to deposited trails, + and – indicate if trails or no trails were visualized, respectively.

Complementation of GAP45-iKO by overexpression of GAP80Ty demonstrates that GAP80 is able to recruit a MyoA motor complex at the pellicle and to maintain sufficient cohesion between the PM and the IMC.

### MyoC-glideosome contributes to invasion

**Figure 8:**
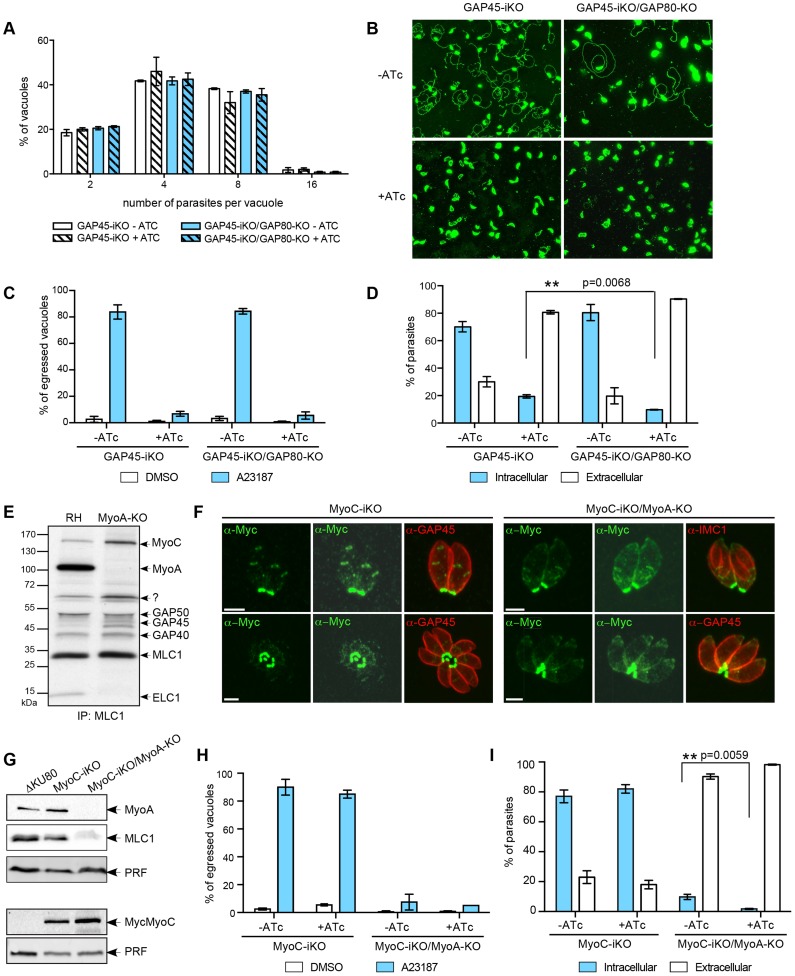
MyoC-glideosome is not involved in cell division but in invasion. A. Intracellular growth assay performed on GAP45-iKO and GAP45-iKO/GAP80-KO strains by determining the number of parasites per vacuole after 48 hours ± ATc. Data are represented as mean ± SD. B. Gliding assay performed on poly-L-lysine coated coverslips with GAP45-iKO and GAP45-iKO/GAP80-KO strains after 42 hours ± ATc. C. Ionophore-induced egress assay of GAP45-iKO and GAP45-iKO/GAP80-KO strains was performed by treating the parasites with DMSO or Ca2+-ionophore A23187 for 5 min after 56 hours ± ATc before The results are expressed as a percentage of ruptured vacuoles and represented as mean ± SD. D. Invasiveness of GAP45-iKO and GAP45-iKO/GAP80-KO strains was determined using a two-color immunofluorescence assay performed after 42 hours ± ATc. Intracellular: invaded parasites, extracellular: attached parasites. Data are represented as mean ± SD. The significance of the data was evaluated using a parametric paired t-test and the two-tailed p-value is written on the graph. E. Co-IP performed on metabolically labeled wild type and MyoA-KO parasites using anti-MLC1 antibodies. F. In MyoA-KO, MycMyoC-iKO relocalized to the periphery of the parasites up to the apical basal ring in addition to its basal localization. Two exposures are presented for MycMyoC localization. Scale bars: 2 µm. G. Western-blot of total extract of MycMyoC-iKO and MycMyoC-iKO/MyoA-iKO analyzed using anti-MyoA, anti-MLC1 and anti-Myc antibodies. The loading control was done at the same time with anti-PRF and fluorescent secondary antibodies on the same membrane as MLC1 for the upper panel and as Myc for the lower panel. H. Ionophore-induced egress assay of MyoC-iKO and MyoC-iKO/MyoA-KO strains performed by treating the parasites with DMSO or Ca2+-ionophore A23187 for 5 min after 54 hours ± ATc before The results are expressed as a percentage of ruptured vacuoles and represented as mean ± SD. I. Red/green invasion assay performed after 42 hours ± ATc. Intracellular: invaded parasites, extracellular: attached parasites. Data are represented as mean ± SD. The significance of the data was evaluated using a parametric paired t-test and the two-tailed p-value is written on the graph.

Ultimately, to circumvent potential compensatory mechanisms due to the plasticity between the MyoA- and MyoC-glideosomes, we opted for the disruption of *GAP80* in the GAP45-iKO background (GAP45-iKO/GAP80-KO). When compared to GAP45-iKO, GAP45-iKO/GAP80-KO did not exhibit any defect in intracellular growth in the presence of ATc demonstrating that the MyoC-glideosome does not play a role in cell division (Figure 8A). In the presence of ATc, GAP45-iKO parasites were not able to egress from infected cells in response to calcium ionophore (A23187) stimulation as monitored by time-lapse microscopy (Videos S3, S4). The same phenotype was observed for GAP45-iKO/GAP80-KO parasites depleted in GAP45 (Videos S5, S6). In agreement with these observations, the two mutants showed severe defects in gliding (Figure 8B) and in egress assays following ATc treatment (Figure 8C and Table 1). Depletion of GAP45 in GAP45-iKO was previously reported to exhibit 20% residual invasion [14] that could be attributed either to leakiness of the Tet-inducible system, to a redundant or compensatory effect via the action of a distinct motor, or a distinct motor-independent mechanism of host cell penetration [17]. Invasion assays performed with GAP45-iKO/GAP80-KO in the presence of ATc revealed an enhanced defect with 50% less invasion compared to GAP45-iKO (Figure 8D and Table 1). These results establish that the MyoC-glideosome contributes to an efficient invasion process in *T. gondii*.

To further investigate the behavior of the MyoC-glideosome in the absence of MyoA, we freshly excised the gene from the loxP-MyoA strain [17] and cloned the parasites. GAP45 and MLC1 were previously shown to remain localized at the periphery of the parasite in the absence of MyoA [17] and hence antibodies raised against these two proteins were used for co-IP experiments to assess the composition of the complex (Figures 8E and S7). The same experiments were performed in parallel with MyoA-iKO parasites depleted in MyoA [12]. In both cases, around 50% more MyoC is found associated with MLC1 and GAP45 in the absence of MyoA (average of 2 and 3 independent experiments for MyoA-KO and MyoA-iKO, respectively, figure S7).

To determine if MyoC could substitute for the absence of MyoA in the peripheral glideosome and be possibly responsible for the residual invasion [17], *MyoA* was disrupted in the MyoC-iKO background by CRISPR/CAS9 mediated gene disruption [28]. Stable parasites were obtained and 3 independent clones were sequenced for the mutations introduced in the *MyoA* locus to repair the double-stranded break generated by the CAS9 at the specific target sequence (Figure S7D). In the absence of MyoA, MyoC is localized not only to the basal polar ring of mature parasites but also relocalized peripherally up to the apical polar ring (Figure 8F). In the absence of MyoA, the amount of MLC1 dropped dramatically while concurrently the amount of MyoC slightly increased (Figure 8G). As MyoA-KO has already a very severe phenotype in egress, no further aggravation could be scored in MyoC-iKO/MyoA-KO treated with ATc (Figure 8H). In contrast, the invasion defect that was around 10% in MyoA-KO further dropped to less than 2% upon MyoC depletion (Figure 8I and Table 1).

## Discussion

**Figure 9:**
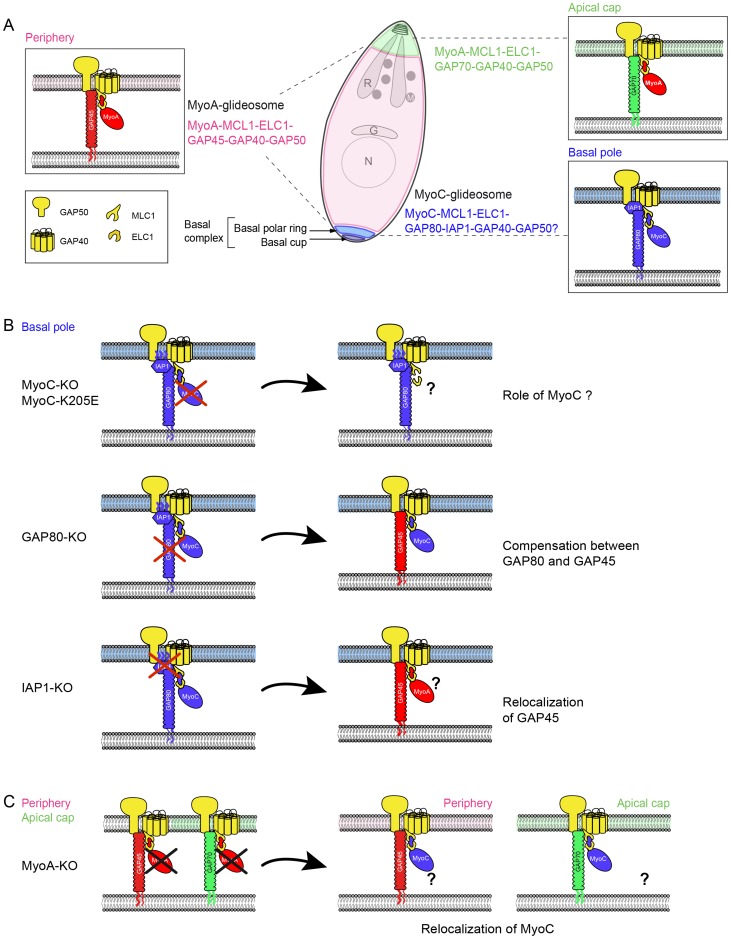
Model of redundancy and compensation mechanisms between the MyoA- and MyoC-glidesome of *Toxoplasma gondii*. A. Localization and composition of the three glideosomes in *T. gondii* tachyzoite. B. Illustration of the composition of the basal glideosome according to the component that has been targeted for deletion. C. Illustration of the composition of the glideosomes upon deletion of MyoA.

This study reports the identification and characterization of a new glideosome in *T. gondii* tachyzoites through the dissection of GAP80, a gliding-associated protein belonging to the GAP45 family and localized to the basal polar ring. The overall arrangement of the three glideosomes is similar and centered around a GAP45 family member that recruits a myosin motor complex to a sub-compartment of the IMC (Figure 9A). GAP45 is conserved across the phylum while GAP70 and GAP80 are restricted to the Coccidian subgroup of Apicomplexa that possess a sub-compartmentalized IMC. The three proteins are predicted to be N-terminally acylated at the plasma membrane and exhibit an extended central region predicted to form a coiled-coil domain or short alpha helices that vary significantly in length. While GAP45 recruits MyoA-MLC1-ELC1 along the central IMC, GAP70 and GAP80 are tailored for the apical cap and the basal complex, respectively. GAP80 recruits MyoC and assembles the MyoC-glideosome that shares GAP50, GAP40, MLC1 and ELC1 with the MyoA-glideosome. Further characterization of the complex identified IAP1, which is the necessary determinant to restrict its localization to the posterior polar ring. Deletion of IAP1 resulted in the loss of MyoC and GAP80 staining at the posterior ring. Given the absence of a TMD, the localization of IAP1 could either be mediated by palmitoylation that would stabilize the protein in the membrane bilayer, or by interaction with an un-identified protein. Alanine substitution of the three N-terminal cysteine residues predicted to be palmitoylated resulted in a cytoplasmic localization of the mutated IAP1. This result strongly suggests that palmitoylation at one or more sites is involved in the attachment of IAP1 to the lowest sub-compartment of the IMC and basal polar ring. In this context, one of the two recently characterized IMC-located protein S-acyl transferases might play an instrumental role in targeting [30]. Interestingly, the truncated version of IAP1 that encompasses 113 aa including three of the four predicted palmitoylated cysteine residues was associated with the basal sub-compartment of the IMC but not anymore to the polar ring. The same relocalization was observed for GAP80 indicating that these two proteins are interacting together and implicates the N-terminus of IAP1. The C-terminus of IAP1 is therefore associated with the polar ring either directly by palmitoylation or possibly by interaction with an integral membrane protein that remains to be identified.

MyoC and MORN1 are the only two proteins identified so far at the posterior polar ring of the growing daughter cells. MORN1 emerges much earlier than MyoC in dividing parasites where is also detected as dots at the extremities of the nascent IMC and at the centrocone [8], [23], [31]. MyoC was anticipated to play a role in cytokinesis by acting in a constrictive ring with MORN1 however no perturbation of MORN1 was observed upon cytochalasin D treatment suggesting already that the driving force of the constriction of the basal ring was unlikely dependent on actin/myosin [31]. Here we show that parasites lacking MyoC still assemble the basal complex as observed by the localization of GAP80 and also divide normally. In contrast, disruption of MORN1 has been achieved using two different strategies that led to a defect in basal complex assembly, cytokinesis and apicoplast segregation [32], [33].

MLC1 and ELC1 are two myosin light chains shared between MyoA and MyoC as shown by their ability to co-immunoprecipitate these two motors (Figure 2). In absence of MyoA, MLC1 remains associated with GAP45 and the pellicle confirming the interaction previously described between the C-terminal part of GAP45 and the N-terminal extension of MLC1 [14] while the signal of ELC1 is largely reduced (Figure 8E).

The fact that GAP70 and GAP45 are assembled with the same components has complicated the assessment of GAP70 function since a compensatory effect via GAP45 could not be excluded [14]. Deletion of GAP80 led to a significant recruitment of MyoC by GAP45. This compensatory mechanism illustrates the versatility of GAP45 and GAP80 in recruiting both motors (Figure 9B). While the three GAP45 family members are expressed in tachyzoites, only GAP45 appears to compensate for the loss of the two others probably due to its high level of expression. Although GAP45 might not be optimally tailored to function at the apical and posterior sub-compartments of the IMC, the compensation in the absence of GAP70 or GAP80 is sufficient to sustain gliding, invasion and egress. In contrast, GAP70 or GAP80 fail to compensate for the absence of GAP45 likely due to structural constraints and low level of their expression. Consistent with this view, the overexpression of a second copy of either GAP70 or GAP80 partially complemented the loss of GAP45 leading to a sub-optimal spacing between the IMC and the PM as shown in the case of GAP70 [14]. To circumvent those compensatory mechanisms, *GAP80* was disrupted in GAP45-iKO. While motility is already severely compromised in GAP45-iKO, an additional 50% decrease in invasion was observed in GAP45-iKO/GAP80-KO compared to GAP45-iKO upon ATc treatment. Given that GAP45 fulfills the dual function of recruiting the MyoA motor complex to the pellicle and holding together the two membranes of the pellicle during motility [14], it was not possible to distinguish the impact of each phenomenon on motility, egress and invasion in GAP45-iKO/GAP80-KO.

MyoC-glideosome harbors two other specific components, MyoC and IAP1 that in principle offer an opportunity to address directly the function of the basal glideosome upon individual deletion of these two genes however significant plasticity and compensatory mechanisms were observed as well (Figure 9B). Deletion of IAP1 led to the disassembly of MyoC-glideosome and its replacement at the basal pole by at least one component of MyoA-glideosome, GAP45, based on IFA. Deletion of MyoC showed no significant impact on the parasite lytic cycle, as also recently reported [18]. However, co-IP experiments performed by immunoprecipitating either GAP80 or IAP1 showed that MyoA can be incorporated into the basal glideosome. Importantly, even a non-functional form of MyoC failed to integrate into the glideosome of the mature posterior pole, offering again the possibility for a compensatory effect to replace the defective MyoC.

The ultimate way to chase MyoC function was to disrupt both *MyoC* and *MyoA* simultaneously. However the recent report of the inability to clone excised-MyoA/MyoC-KO parasites [18] was indicative of a synthetic lethality between the two genes. While as expected, we also failed to generate parasites lacking both genes, we however succeeded in generating a MyoC-iKO/MyoA-KO. Importantly, in the absence of MyoA, MyoC clearly relocalized to the parasite periphery and at the apical pole, providing evidence that MyoC could partially replace MyoA in the peripheral and apical glideosomes (Figure 9C). Upon depletion of MyoC with ATc, invasion dropped to less than 2% compared to wild type parental strain, which further confirmed the central role played by both motors in invasion.

## Materials and Methods

### Preparation of *T. gondii* genomic DNA and RNA

Genomic DNA has been prepared from tachyzoites (RH strain) using the Wizard SV genomic DNA purification system (Promega). RNA was isolated from tachyzoites using Trizol (Invitrogen). Total cDNA was then generated by RT-PCR performed with the Superscript II reverse transcriptase (Invitrogen) according to the manufacturer's instructions.

### Cloning of DNA constructs

All amplifications were performed with the LA or Ex Taq (TaKaRa) polymerases and the primers used are listed in supplementary table S4.

#### pTUB8GAP70TyCtGAP80, pTUB8MycHisGFPCtGAP80 and pTUB8GAP80Ty

To create pTUB8GAP70TyCtGAP80 and pTUB8MycHisGFPCtGAP80, the C-terminal part of *GAP80* (TGME49_246940, aa 309 to 393) was amplified from cDNA with primers GAP80-1/GAP80-2, digested with *NsiI* and *Pac*I and cloned between *PstI* and *Pac*I sites of the pTUB8TyGAP70 vector and between *NsiI* and *Pac*I sites of pTUB8MycHisGFPCAP45 vector [14]. To create pTUB8GAP80Ty, the aa 1 to 308 fragment of GAP80 was amplified from cDNA with primers GAP80-3/GAP80-4, digested with *Eco*RI and *Nsi*I and cloned into pTUB8GAP70TyCtGAP80 between the same restriction sites.

#### pTUB8-IAP1Ty and pTUB8-AAA-IAP1Ty

The ORF of IAP1 (TGME49_283510) has been amplified from cDNA with primers IAP1-1/IAP1-2, digested with *Eco*RI and *Nsi*I and cloned into pTUB8MycGFPPfMyoAtailTy-HX [11] between the same restriction sites. Site directed mutagenesis was performed by amplifying the ORF of IAP1 with primers IAP1-3/IAP1-2. The fragment displaying the 3 first cysteines mutated to alanine residues was then digested with *Eco*RI and *Nsi*I and cloned into pTUB8MycGFPPfMyoAtailTy-HX [11] between the same restriction sites.

#### Knock-in of GAP70 and GAP80 (pKI-GAP70Ty and pKI-GAP80Ty)

A genomic fragment of *GAP70* was amplified with primers GAP70-1/GAP70-2, digested with *Kpn*I and *Nsi*I and cloned into the same sites of the pTUB8TyGAP70 vector [14]. *Sph*I was then used to linearize the plasmid before transfection. The knock-in construct of *GAP80* was derived from pTUB8GAP80Ty. The promoter region was removed by digestion with *Kpn*I and *Xho*I restriction enzymes. The T4 DNA polymerase (NEB) was then used to blunt the vector before ligation to re-circularize the plasmid. Finally, the plasmid was linearized with *Hind*III for transfection.

#### Knockout of GAP80 (p2854-DHFR-5′3′GAP80, pT/230-ble-5′3′GAP80)

Around 3 kb of the 5′ and 3′ flanking regions of *GAP80* were amplified by PCR with primers GAP80-5/GAP80-6 and GAP80-7/GAP80-8, respectively. The 5′ flanking region was then digested and cloned into the *Hind*III restriction site of the p2854-DHRF [34] and pT/230-ble vector [35], respectively and the 3′ flanking region into the *Not*I site. A *Mfe*I site was introduced in primers GAP80-5 and GAP80-8 to digest the plasmid before transfection.

#### Knock-in of MyoC, ELC1, IAP1 and ILP1 (pKI-MyoC-3Ty, pKI-ELC1-3Ty, pKI-IAP1-3Ty and pKI-ILP1-3Ty)

Genomic fragments of *MyoC* (TGME49_255190), *ELC1* (TGME49_269442), *IAP1* (TGME49_283510) and *ILP1* (TGME49_113380) were amplified by PCR using the primers MyoC-1/MyoC-2, ELC1-1/ELC1-2, IAP1-4/IAP1-2 and ILP-1/ILP-2 respectively, then digested with *Kpn*I and *Nsi*I restriction enzymes and cloned into the same sites of the pTUB8MIC13-3Ty-HX [36] to introduce 3 Ty-tags at the C-termini. Before transfection, pKI-MyoC-3Ty, pKI-ELC1-3Ty, pKI-IAP1-3Ty and *pKI-ILP1-3Ty* were linearized with *Xcm*I, *Pst*I, *Bgl*II and *Eco*RI, respectively.

#### Knock-in of NT-IAP1 (pLIC-NT-IAP1-3Myc)

A truncation of the endogenous IAP1 (aa 1 to 113) was created by knock-in. A genomic fragment corresponding to the N-terminal part of *IAP1* was amplified by PCR using the primers IAP1-5/IAP1-6 and inserted into the pLIC-3Myc_DHFR vector (from Dr V. Carruthers, [20]) using the ligation independent cloning strategy [37].

#### Knockout of IAP1 (p2854-DHFR-5′3′IAP1)

Around 1.5 kb of the 5′ and 3′ flanking regions of *IAP1* were amplified by PCR with primers IAP1-7/IAP1-8 and IAP1-9/IAP1-10, respectively. The 5′ flanking region was then cloned between the *Apa*I and *Hind*III restriction sites of the p2854-DHRF [34] and the 3′ flanking region between the *Xba*I and *Not*I sites. A *SbfI* site was introduced in primers IAP1-7 and IAP1-10 to digest the plasmid before transfection.

#### MyoC-iKO (5′MyoC-pTetO7Sag4-MycNtMyoC)

A 2.5 kb genomic DNA fragment of the 5' flanking region of *MyoC* was amplified by PCR with primers MyoC-5/MyoC-6 and cloned into the *Apa*I site of the pTub5CAT vector. A 2 kb genomic DNA fragment corresponding to the N-terminal sequence of *MyoC* was amplified by PCR with primers MyoC-3/MyoC-4 and cloned into *Nsi*I and *Bam*HI restriction sites of ptetO7Sag4MycGFP vector [12]. Finally, the pTetO7Sag4-MycNtMyoC fragment was subcloned into the *Sac*I site of pTub5CAT. A *Pme*I restriction site was introduce in MyoC-4 and MyoC-6 primers to digest the plasmid before transfection in the TATi line [12].

#### MycMyoC-K205E (5′MyoC-pTetO7Sag4-MycNtMyoC- K205E)

The same cloning strategy as for MycMyoC-WT was used, except that the pTetO7Sag4-MycNtMyoC-WT vector was modified as follow before subcloning into the pTub5CAT. A synthetic fragment corresponding to the MyoC cDNA (aa 1 to 238) with the ATP-binding site (GESGAGKT) mutated to GESGAGET was generated by Genscript with the human codon usage and cloned in the pTetO7Sag4-MycNtMyoC-WT vector between the EcoRI and StuI sites. This plasmid was digested with *Pme*I before transfection in the Ku80-KO line [19], [20].

#### MyoC-head endogenous copy (pLIC- MyoC-ΔN&T-Myc)

A truncated version of MyoC (aa 1 to 760) was generated by introducing 3 Myc-tags after the head domain in the endogenous locus by knock-in. For this, a genomic DNA fragment was amplified by PCR with primers MyoC-7/MyoC-8 and inserted into the pLIC-3Myc_DHFR vector (from Dr V. Carruthers, [20]) using the ligation independent cloning strategy [37].

#### Knockout of MyoC (pTub5-CAT-5′3′MyoC)

Around 1.6 kb of the 5′ and 3′ flanking regions of *MyoC* were amplified by PCR with primers MyoC-9/MyoC-10 and MyoC-11/MyoC-12, respectively. The 5′ flanking region was then cloned between the *Kpn*I and *Hind*III restriction sites of the pTub5-CAT and the 3′ flanking region between the *Bam*HI and *Not*I sites. The plasmid was cut with *Kpn*I and *Not*I restriction enzymes before transfection.

#### MyoA- and MyoC-specific CRISPR/CAS9 plasmids

These two vectors have been generated using the Q5 site-directed mutagenesis kit (New England Biolabs) with the vector pSAG1::CAS9-U6::sgUPRT as template [28] (a generous gift from Dr L.D. Sibley). The UPRT-targeting gRNA was replace by the MyoA and MyoC specific gRNA using the primer pairs gRNA-MyoA/gRNA-rev and gRNA-MyoC/gRNA-rev, respectively.

### T. gondii culture

*T. gondii* tachyzoites (RH*hxgprt^-^*, *ku80-ko-hxgprt^-^* [20] strains and their derivatives expressing the epitope-tagged proteins) were grown in confluent human foreskin fibroblasts (HFF) or in Vero cells maintained in Dulbecco's Modified Eagle's Medium (DMEM, life technology, Invitrogen) supplemented with 10% fetal calf serum, 2 mM glutamine and 25 µg/ml gentamicin.

### Parasite transfection and selection of stable transformants

Parasite transfections were performed by electroporation as previously described [38]. The *hxgprt* gene was used as a positive selectable marker in the presence of mycophenolic acid (25 mg/mL) and xanthine (50 mg/mL) for the pTUB8 vectors transfected in RH strains, as described previously [39].

Ku80-KO [19], [20] and derivative strains have been transfected with 20 µg of the knock-in constructs and with 40 and 60 µg of p2854-DHFR-5′3′GAP80, p2854-DHFR-5′3′IAP1 or pTub5-CAT-5′3′MyoC vectors. 1 µg/ml of pyrimethamine and 20 µM of chloramphenicol have been used to select the resistant parasites.

The pT/230-ble-5′3′GAP80 and the pTUB8GAP80Ty vectors were transfected in the ΔGAP45e/GAP45i strain [14]. Knockout parasites transfected with pT/230-ble-5′3′GAP80 were selected with 30 mg/ml of phleomycin and complemented parasites transfected with pTUB8GAP80Ty were selected with 1 µg/ml of anhydrotetracyclin (ATc).

To facilitate insertion by double homologous recombination in the MyoC locus, 70 ug of the 5′MyoC-pTetO7Sag4-MycNtMyoC-mut vector, and 30 ug of the MyoC gRNA-specific CRISPR/CAS9 vector have been transfected. To efficiently disrupt MyoA locus, 30 ug of the MyoA gRNA-specific CRISPR/CAS9 vector have been transfected. In both cases, 24 hours after transfections, parasites were sorted by flow cytometry and cloned into 96-well plates using a Moflo Astrios (Beckman Coulter).

### Antibodies

The antibodies used in these study were described previously as follow: polyclonal rabbit: α-catalase [40], α-GAP45, α-PRF [41], α-MyoA [42], α-MLC1 [11], and α-GAP40 [21]; mouse monoclonal, α-ACT [11], α-Ty (BB2), α-Myc (9E10). For Western blot analyses, secondary peroxidase conjugated goat α-rabbit/mouse antibodies (Molecular Probes) were used. For immunofluorescence analyses, the secondary antibodies Alexa Fluor 488 and Alexa Fluor 594-conjugated goat α-mouse/rabbit antibodies (Molecular Probes) were used.

To generate the α-IMC1, a fragment encoding amino acids 140–610 was amplified from RH cDNA with primers IMC1-1/IMC1-2 and cloned into pETHTb (kindly provided by A. Houdusse, Paris) between the *Bam*HI and *Xho*I sites in frame with 6 N-terminal histidine residues. The fusion protein was expressed into *E. coli* BL21 strain, affinity purified on Ni-NTA-agarose beads (Qiagen) according to the manufacturer's protocol under nature conditions and used to immunize two rabbits according to the Eurogentec standard protocol.

### Immunofluorescence assay (IFA) and confocal microscopy

Parasite-infected HFF cells seeded on cover slips were fixed with 4% paraformaldehyde (PFA) or 4% PFA/0.05% glutaraldehyde (PFA/GA) in PBS, depending of the antigen to be labeled. Fixed cells were then processed as previously described [42]. Confocal images were generated with two laser scanning confocal microscopes: a Leica (TCS-NT DM/IRB and SP2) using a 1003 Plan-Apo objective with NA 1.4 and a Zeiss (LSM700, objective apochromat 63x/1.4 oil) at the Bioimaging core facility of the Faculty of Medicine, University of Geneva. Stacks of sections were processed with ImageJ and projected using the maximum projection tool.

### Subcellular fractionations

Freshly released tachyzoites were harvested, washed in PBS, and then resuspended in PBS, PBS/1% Triton X-100, PBS/1M NaCl, or PBS/0.1 M Na2CO3 [pH 11.5]. Parasites were lysed by freeze and thaw followed by sonication on ice. Pellet and soluble fractions were separated by centrifugation for 30 minutes at 14,000 rpm at 4°C. The solubility of the catalase (CAT) was checked in the different conditions as control.

### Western blot analyses

Parasites were lysed in PBS or PBS-1% Triton X-100 and mixed with SDS–PAGE loading buffer under reducing conditions. The suspension was either boiled or subjected to sonication on ice. SDS-PAGE was performed using standard methods. Separated proteins were transferred to nitrocellulose membranes and probed with appropriate antibodies in 5% non-fat milk powder in PBS-0.05% Tween20. Bound secondary peroxidase conjugated antibodies were visualized using either the ECL system (GE healthcare) or SuperSignal (Pierce).

### Plaque assay

Host cells were infected with parasites for 6 or 7 days before fixation with PFA/GA. Giemsa staining was then performed as described in Plattner *et al.*, 2008.

### Intracellular growth assay

Parasites were grown for 24 hours before fixation with PFA/GA. Double-labelling IFA was performed using α-GAP45 and α-actin antibodies. The number of parasites per vacuole was determined by counting the parasites in 100 vacuoles in duplicate for three independent experiments. For GAP45-iKO and MyoC-iKO containing strains, parasites were pre-treated for 30 hours ± ATc prior to inoculation.

### Red/green invasion assay

This assay was performed as previously described [43] with the following specificities. Freshly released parasites were then inoculated on new host cells and allowed to invade for 20 minutes before fixation with PFA/GA for 5 minutes. The samples were first incubated with anti-SAG1 antibodies in PBS-2% BSA to reveal extracellular parasites and then, following Triton X-100 permeabilization, they were incubated with anti-IMC1 or anti-GAP45 antibodies to reveal the intracellular parasites. The number of intracellular and extracellular parasites was determined by counting 100 parasites in duplicate for four independent experiments. For GAP45-iKO and MyoC-iKO containing strains, parasites were pre-treated for 42 hours ± ATc.

### Induced egress assay

New host cells were inoculated with freshly released parasites allowed to grow for 30 hours ± ATc. Parasite-infected host cells were then incubated for 5 min at 37°C with DMEM containing 0.06% DMSO or 3 µM of the Ca^2+^ ionophore A23187 from *Streptomyces chartreusensis* (calbiochem) before fixation. Double-labelling IFA was performed using α-GRA3 and α-SAG1 antibodies. The average number of egressed vacuoles was determined by counting 100 vacuoles for each condition for four independent experiments. For GAP45-iKO and MyoC-iKO containing strains, parasites were pre-treated for 24 hours ± ATc prior inoculation.

### Gliding assay

Parasites were grown for 42 hours ± ATc. Freshly released parasites were allow to settle on poly-L-lysine coated coverslips for 8 minutes in DMEM and then incubated for 10 min in an HEPES/calcium-saline solution before fixation with PFA/GA. Anti-SAG1 antibody was used without permeabilization to visualize the trails and the parasites. Three independent experiments have been performed.

### Metabolic labeling and co-immunoprecipitation (co-IP)

HFF cells were heavily infected with freshly egressed parasites and washed several hours later. After 30 hours, cells were incubated in methionine/cysteine-free DMEM (sigma) for 1 hour before incubation in DMEM containing 50 µCi [^35^S]-labeled methionine/cysteine (Hartmann analytic GmbH) per ml for 4 hours at 37°C. For co-IPs, freshly released tachyzoites were harvested, washed in PBS and lysed in CoIP buffer (1% (v/v) Triton X-100, 50 mM Tris-HCl, pH 8, 150 mM NaCl) in the presence of a protease inhibitor cocktail (Roche). Cells were frozen and thawed five times, sonicated on ice, incubated for 10 min on ice, and centrifuged at 14,000 rpm for 1 hour at 4°C. Supernatants were incubated with monoclonal α-Ty or α-Myc or polyclonal α-GAP45 or α-MLC1 antibodies for 1 hour at 4°C on a rotating wheel. Protein A-Sepharose CL-4B (GE Healthcare Life Sciences) was then added and the incubation continued for 1 hour. Complexes were then washed three times in CoIP buffer. Finally, beads were resuspended in SDS loading buffer under reducing conditions.

### Mass spectrometry

Samples obtained after co-IP assays were separated by SDS-PAGE and stained with coomassie blue or silver stain. Bands of interest were excised from the gel and sent to the Proteomics Core Facility (Faculty of Medicine, Geneva, Switzerland) for analysis according to their standard protocols for protein identification. The fragments were generated with trypsin and the peaklist files were searched against the *Toxoplasma gondii* GT1 database (Toxoplasma Genomics Resource, release 8.2 of 31-May-2013, 8102 entries) using Mascot (Matrix Sciences, London, UK).

### Accession numbers

Genbank accession numbers: KF897514 for TgGAP80 and KF897515 for TgIAP1

## Supporting Information

Figure S1 **GAP45-related proteins.** A. Multiple alignments of the *T. gondii* and *Neospora caninum* GAP45-related protein sequences performed with CLUSTAL W [44]. Identical residues are in red, strongly similar residues in green and weakly similar residues in blue. The myristoylated glycine 2 and palmitoylated cysteine were predicted using myristoylator [45] and CSS-Palm 3.0 [26], respectively and are indicated by a red arrow. The coiled-coil domain predicted with Coils [46] in GAP45 and GAP70 proteins is indicated by a blue spring and the conserved C-terminal part is depicted by a green box. Accession numbers from EupathDB [47]: TgGAP45 (TGME49_223940), NcGAP45 (NCLIV_048570), TgGAP70 (TGME49_233030), NcGAP70 (NCLIV_032850), TgGAP80 (TGME49_246940) and NcGAP80 (NCLIV_063610). B. Schemes of TgGAP45, TgGAP70 and TgGAP80 showing on the left the coiled-coil domains according to the *in silico* prediction performed with coils and on the right the α-helices prediction resulting from the consensus of 8 methods (see supplementary materials and methods). C. Scheme of the knock-in strategy used to introduce a Ty-tag in the endogenous loci of *gap70* or *gap80*.
(PDF)

Figure S2 **MLC1 is shared between two myosin heavy chain complexes.** A. Bound fractions of co-IP experiments performed using anti-Ty antibodies on ^35^S-methionine/cysteine metabolically labeled parasites expressing KI-GAP70Ty and KI-GAP80Ty have been analyzed by western blot with anti-MLC1, anti-MyoA and anti-GAP40 antibodies. B. Parasites stably expressing MLC1Ty were used to perform a co-IP with anti-Ty antibodies. Elution was loaded on a SDS-page gel and stained with Coomassie blue. The bands around 72 and 130 kDa were cut out for analysis by mass spectrometry (results in table S1). The identified proteins are written in red. C. The presence of the MyoC in the bound fraction of co-IP performed by pulling down KI-GAP70Ty and KI-GAP80Ty complexes with anti-Ty antibodies was confirmed using anti-MyoC antibodies [23]. The asterisks indicate the Ig heavy and light chains that cross-react with the antibodies. D. The MycGFPGAP80CT construct that localizes at basal end has been transiently transfected into the KI-MyoC-3Ty and the KI-IAP1-3Ty strains in order to show that these three proteins are located at the same place. Scale bars: 2 µm.
(PDF)

Figure S3 **A basal IMC-associated protein (IAP1) anchors the MyoC-glideosome.** A. Preparative gels of the co-IPs performed with anti-Myc antibodies on lysates of MycGFPCTGAP70 and MycGFPCTGAP80 expressing parasites. The arrows point the band analyzed by mass spectrometry and the asterisks indicate the Ig. B. Multiple alignment of the *T. gondii*, *N. caninum* and *Eimeria tenella* IAP1 sequences performed with CLUSTAL W [44]. Identical residues are in red, strongly similar residues in green and weakly similar residues in blue. The palmitoylated cysteine were predicted using CSS-Palm 3.0 [26], and are highlighted in yellow. The red arrow indicates the end of truncated version generated (KI-NT-IAP1-3Myc). Accession numbers are from EupathDB [47]. C–D. Total extracts of parasites expressing a second copy of IAP1-Ty (C) or an endogenously tagged IAP1 and ILP1 (D) were subjected to western blot analysis performed with anti-Ty antibodies. E. Autoradiograph of the bound fractions obtained after Co-IP performed with anti-Ty antibodies on metabolic labeled parasites expressing KI-GAP80Ty and KI-IAP1-3Ty. The presence of MyoC, MyoA, GAP40 and MLC1 is visible.
(PDF)

Figure S4 **Generation of MycMyoC-iKO and MycMyoC-K205E cell lines.** A. Schematic representation of MyoC highlighting the head (ATPase and actin-binding activities), the IQ-containing neck and the tail domains (left panel) and of the truncated version of MyoC lacking the neck and tail domains (right panel). B–C. Schematic representation of the strategy used to replace the endogenous promoter of MyoC by an inducible promoter (TetO7Sag4) in the TATi background (B) and to introduce a mutated ATP-binding site in the Ku80-KO background (C). The wild type and modified loci are depicted with the position of the primers used to confirm the integration and the expected size of the PCR products. D. PCRs performed on gDNA extracted from TATi, MycMyoC-iKO and MycMyoC-K205E strains to confirm the integrations. The sequences of the primers can be found in the supplementary table S5. E. Intracellular growth assay fixed 24 hours post-invasion. The number of parasites per vacuole were determined and represented as mean +/− SD. F. Invasion assay performed using a two-color immunofluorescence. Intracellular: invaded parasites, extracellular: attached parasites. Data are represented as mean ± SD. G. Calcium ionophore-induced egress assay performed after 30 hours, expressed as a percentage of egressed vacuoles and represented as mean +/− SD.
(PDF)

Figure S5 **Analyses of MyoC-KO and GAP80-KO strains.** A. Intracellular growth assay performed with MyoC-ΔN&T-3Myc and Ku80-KO strains and fixed after 24 hours. The number of parasites per vacuole were determined and represented as mean +/− SD. B. Calcium ionophore-induced egress assay performed with MyoC-ΔN&T-3Myc and Ku80-KO after 30 hours. Data are expressed as a percentage of egressed vacuoles and represented as mean +/− SD. C. Schematic representation of the endogenous and modified locus of *MyoC* in KI-GAP80Ty and MyoC-KO/KI-GAP80Ty strains, respectively. The position of the primers used to confirm the integration and the length of the PCR products are indicated. D. PCRs performed on gDNA extracted from KI-GAP80Ty and MyoC-KO/KI-GAP80Ty strains to confirm the disruption of the *MyoC* gene. end.: endogenous, mod.: modified, CAT: chloramphenicol acetyltransferase gene conferring resistance to chloramphenicol. The sequences of the primers can be found in the supplementary table S5. The star indicates unspecific bands. E. Schematic representation of the endogenous and modified locus of *GAP80* in Ku80-KO strain. The position of the primers used to confirm the integration and the length of the PCR products are indicated. F. PCRs performed on gDNA extracted from Ku80-KO and GAP80-KO strains to confirm the disruption of the *GAP80* gene. end.: endogenous, mod.: modified, DHFR: dihydrofolate reductase gene conferring resistance to pyrimethamine. The sequences of the primers can be found in the supplementary table S5. G. Intracellular growth assay performed with Ku80-KO and GAP80-KO and fixed after 24 hours. The number of parasites per vacuole were determined and represented as mean +/− SD. H. Calcium ionophore-induced egress assay performed with Ku80-KO and GAP80-KO after 30 hours, expressed as a percentage of egressed vacuoles and represented as mean +/− SD.
(PDF)

Figure S6 **Analyses of IAP1-KO strain.** A. Schematic representation of the endogenous and modified locus of *IAP1* in KI-MycMyoC or in KI-GAP80Ty strains. The position of the primers used to confirm the integration and the length of the PCR products are indicated. B. PCRs performed on gDNA extracted from Ku80-KO and IAP1-KO strains to confirm the disruption of the *IAP1* gene. The sequences of the primers can be found in the supplementary table S5. C. Intracellular growth assay performed with Ku80-KO and IAP1-KO and fixed after 24 hours. The number of parasites per vacuole were determined and represented as mean +/− SD. D. Calcium ionophore-induced egress assay performed with Ku80-KO and IAP1-KO after 30 hours, expressed as a percentage of egressed vacuoles and represented as mean +/− SD.
(PDF)

Figure S7 **More MyoC associates with MyoA-glideosome in absence of MyoA.** A–B. Autoradiograph of the bound fractions obtained after Co-IP performed with anti-MLC1 or anti-GAP45 antibodies on metabolic labeled MyoA-iKO parasites treated or not with ATc (A) or RH and MyoA-KO (B). C. Quantification of MyoA and MyoC co-immunoprecipitated with anti-MLC1 antibodies has been performed with ImageJ (see supplementary materials and methods) from 3 independent experiments performed with MyoA-iKO strain and 2 independent experiments performed with MyoA-KO. Data are represented as mean +/−. D. Sequencing of 3 independent clones to confirm the disruption of MyoA induced by CAS9. The gRNA used is written in red.
(PDF)

Table S1 **Identification of MyoC and GAP80 by mass spectrometry after co-IP performed with anti-Ty antibodies on parasites stably expressing TgMLC1Ty.**
(PDF)

Table S2 **List of the proteins identified by mass spectrometry from co-IP performed with anti-Myc antibodies on parasites stably expressing MycGFPCtGAP80 as a second copy.**
(PDF)

Table S3 **Identification of IAP1 by mass spectrometry after co-IP performed with anti-Myc antibodies on parasites stably expressing MycGFPCtGAP80.**
(PDF)

Table S4 **Oligonucleotide primers used in this study for cloning.**
(PDF)

Table S5 **Oligonucleotide primers used in this study for PCR analyses.**
(PDF)

File S1 **Supplementary materials and methods.**
(DOCX)

Video S1 **Induced egress assay of wild type (WT) parasites recorded at two frames per second and played at 50 frames per second.** Time is indicated in minutes: seconds and scale bar represent 10 µm.
(MOV)

Video S2 **Induced egress assay of MyoC-KO parasites recorded at two frames per second and played at 50 frames per second.** Time is indicated in minutes: seconds and scale bar represent 10 µm.
(MOV)

Video S3 **Induced egress assay of GAP45-iKO parasites in absence of ATc recorded at two frames per second and played at 50 frames per second.** Time is indicated in minutes: seconds and scale bar represent 10 µm.
(MOV)

Video S4 **Induced egress assay of GAP45-iKO parasites in presence of ATc recorded at two frames per second and played at 50 frames per second.** Time is indicated in minutes: seconds and scale bar represent 10 µm.
(MOV)

Video S5 **Induced egress assay of GAP45-iKO/GAP80-KO parasites in absence of ATc recorded at two frames per second and played at 50 frames per second.** Time is indicated in minutes: seconds and scale bar represent 10 µm.
(MOV)

Video S6 **Induced egress assay of GAP45-iKO/GAP80-KO parasites in presence of ATc recorded at two frames per second and played at 50 frames per second.** Time is indicated in minutes: seconds and scale bar represent 10 µm.
(MOV)
