# Supplementary figures and images for: Plasticity between MyoC- and MyoA-Glideosomes: An Example of Functional Compensation in Toxoplasma gondii Invasion
Source: PLoS Pathog. 2014 Nov 13;10(11):e1004504. doi: 10.1371/journal.ppat.1004504 (PMC4231161; doi:10.1371/journal.ppat.1004504)

Supplementary Figure S1

A

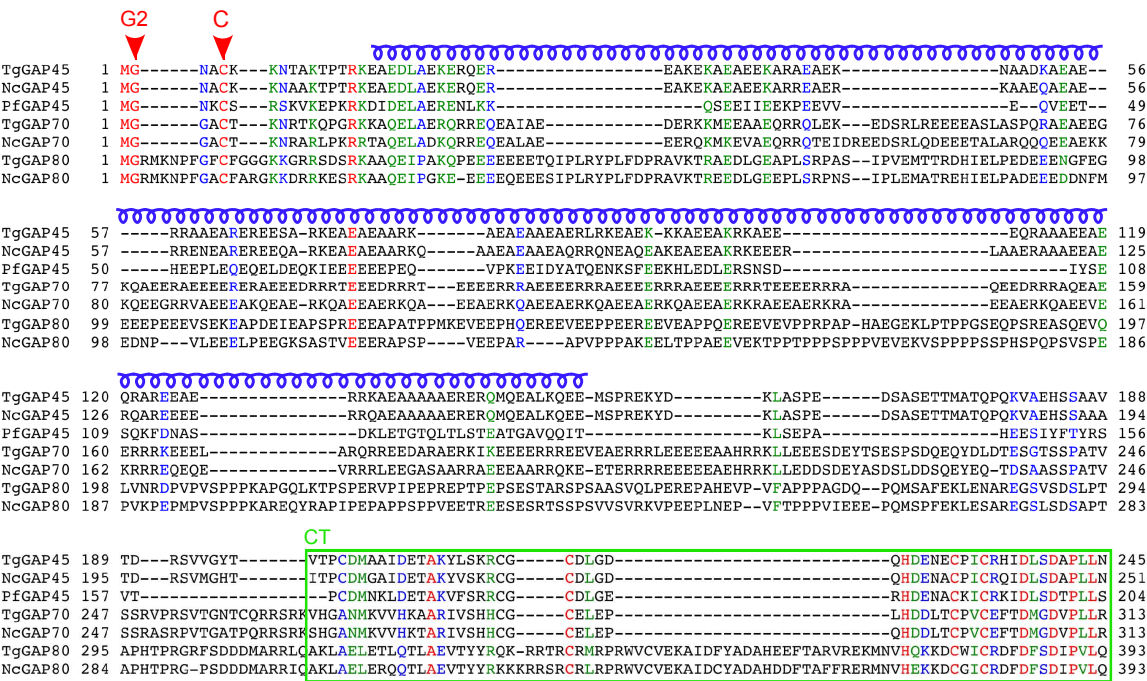

B

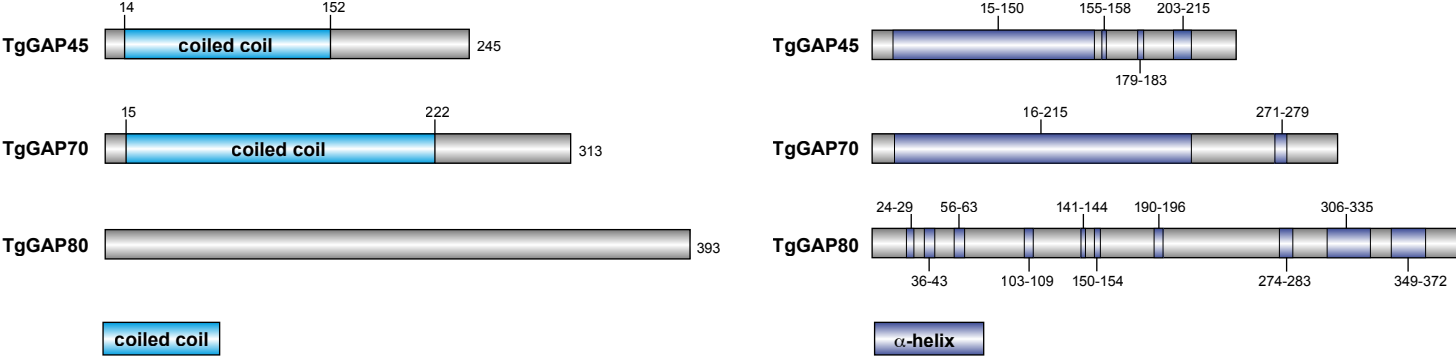

C

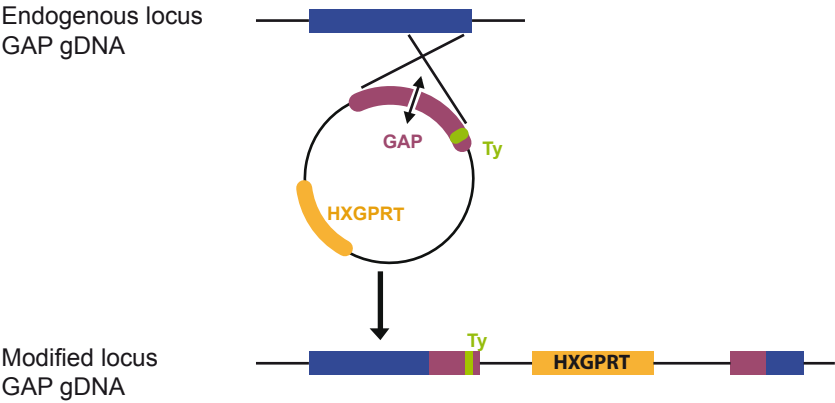

Supplement: Figure S1 — GAP45-related proteins. A. Multiple alignments of the T. gondii and Neospora caninum GAP45-related protein sequences performed with CLUSTAL W [44]. Identical residues are in red, strongly similar residues in green and weakly similar residues in blue. The myristoylated glycine 2 and palmitoylated cysteine were predicted using myristoylator [45] and CSS-Palm 3.0 [26], respectively and are indicated by a red arrow. The coiled-coil domain predicted with Coils [46] in GAP45 and GAP70 proteins is indicated by a blue spring and the conserved C-terminal part is depicted by a green box. Accession numbers from EupathDB [47]: TgGAP45 (TGME49_223940), NcGAP45 (NCLIV_048570), TgGAP70 (TGME49_233030), NcGAP70 (NCLIV_032850), TgGAP80 (TGME49_246940) and NcGAP80 (NCLIV_063610). B. Schemes of TgGAP45, TgGAP70 and TgGAP80 showing on the left the coiled-coil domains according to the in silico prediction performed with coils and on the right the α-helices prediction resulting from the consensus of 8 methods (see supplementary materials and methods). C. Scheme of the knock-in strategy used to introduce a Ty-tag in the endogenous loci of gap70 or gap80. (PDF) [file ppat.1004504.s001.pdf]

Supplementary figure S2

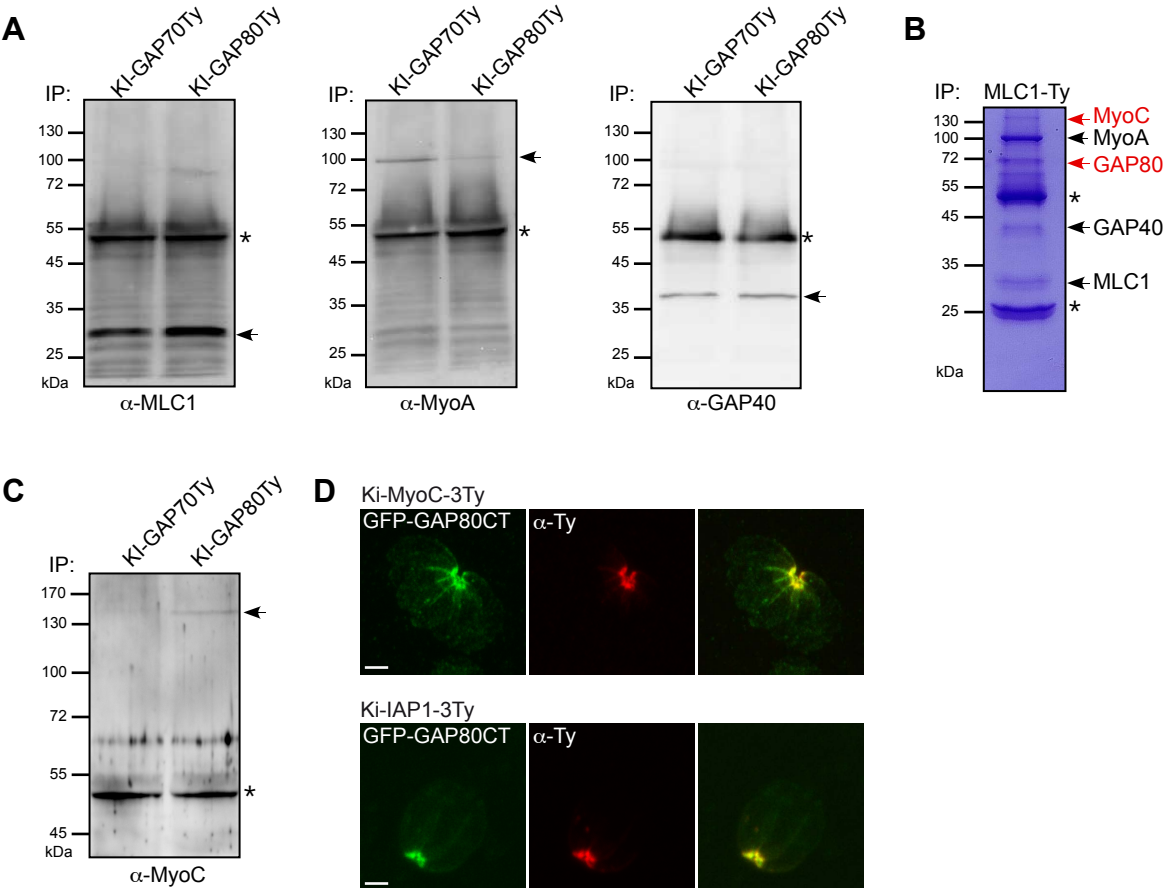

Supplement: Figure S2 — MLC1 is shared between two myosin heavy chain complexes. A. Bound fractions of co-IP experiments performed using anti-Ty antibodies on 35S-methionine/cysteine metabolically labeled parasites expressing KI-GAP70Ty and KI-GAP80Ty have been analyzed by western blot with anti-MLC1, anti-MyoA and anti-GAP40 antibodies. B. Parasites stably expressing MLC1Ty were used to perform a co-IP with anti-Ty antibodies. Elution was loaded on a SDS-page gel and stained with Coomassie blue. The bands around 72 and 130 kDa were cut out for analysis by mass spectrometry (results in table S1). The identified proteins are written in red. C. The presence of the MyoC in the bound fraction of co-IP performed by pulling down KI-GAP70Ty and KI-GAP80Ty complexes with anti-Ty antibodies was confirmed using anti-MyoC antibodies [23]. The asterisks indicate the Ig heavy and light chains that cross-react with the antibodies. D. The MycGFPGAP80CT construct that localizes at basal end has been transiently transfected into the KI-MyoC-3Ty and the KI-IAP1-3Ty strains in order to show that these three proteins are located at the same place. Scale bars: 2 µm. (PDF) [file ppat.1004504.s002.pdf]

Supplementary figure S3

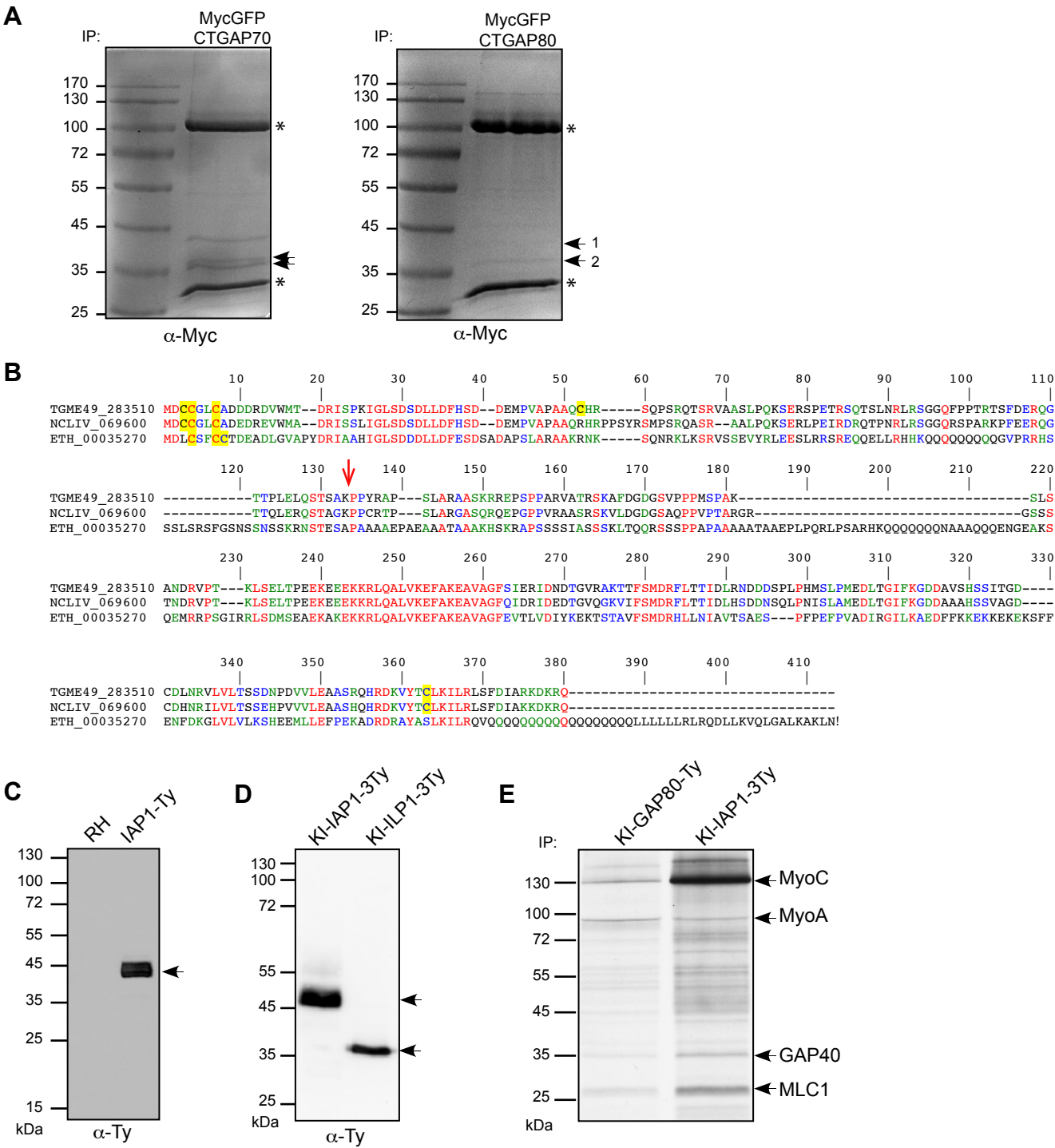

Supplement: Figure S3 — A basal IMC-associated protein (IAP1) anchors the MyoC-glideosome. A. Preparative gels of the co-IPs performed with anti-Myc antibodies on lysates of MycGFPCTGAP70 and MycGFPCTGAP80 expressing parasites. The arrows point the band analyzed by mass spectrometry and the asterisks indicate the Ig. B. Multiple alignment of the T. gondii, N. caninum and Eimeria tenella IAP1 sequences performed with CLUSTAL W [44]. Identical residues are in red, strongly similar residues in green and weakly similar residues in blue. The palmitoylated cysteine were predicted using CSS-Palm 3.0 [26], and are highlighted in yellow. The red arrow indicates the end of truncated version generated (KI-NT-IAP1-3Myc). Accession numbers are from EupathDB [47]. C–D. Total extracts of parasites expressing a second copy of IAP1-Ty (C) or an endogenously tagged IAP1 and ILP1 (D) were subjected to western blot analysis performed with anti-Ty antibodies. E. Autoradiograph of the bound fractions obtained after Co-IP performed with anti-Ty antibodies on metabolic labeled parasites expressing KI-GAP80Ty and KI-IAP1-3Ty. The presence of MyoC, MyoA, GAP40 and MLC1 is visible. (PDF) [file ppat.1004504.s003.pdf]

## Supplementary figure S4

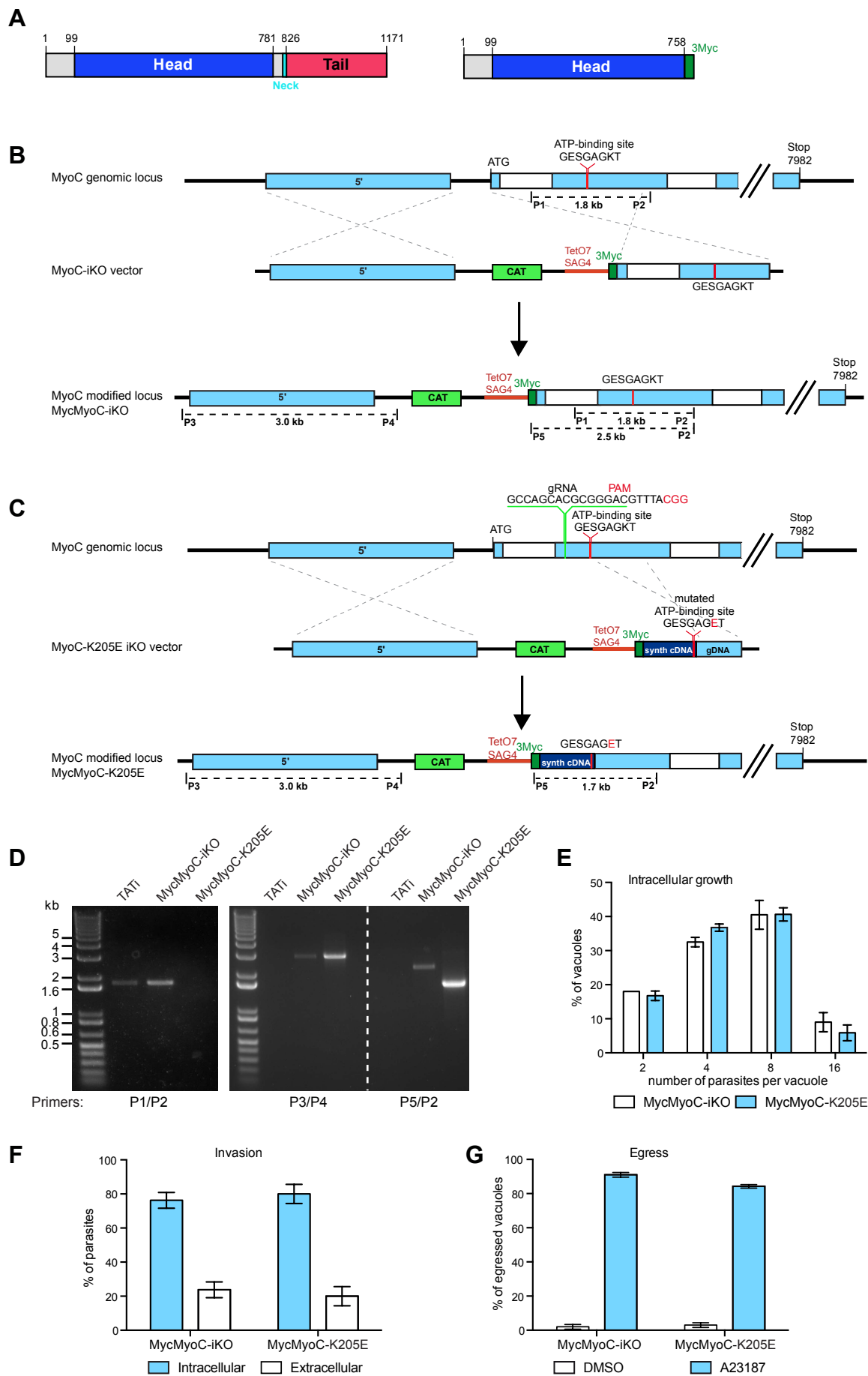

Supplement: Figure S4 — Generation of MycMyoC-iKO and MycMyoC-K205E cell lines. A. Schematic representation of MyoC highlighting the head (ATPase and actin-binding activities), the IQ-containing neck and the tail domains (left panel) and of the truncated version of MyoC lacking the neck and tail domains (right panel). B–C. Schematic representation of the strategy used to replace the endogenous promoter of MyoC by an inducible promoter (TetO7Sag4) in the TATi background (B) and to introduce a mutated ATP-binding site in the Ku80-KO background (C). The wild type and modified loci are depicted with the position of the primers used to confirm the integration and the expected size of the PCR products. D. PCRs performed on gDNA extracted from TATi, MycMyoC-iKO and MycMyoC-K205E strains to confirm the integrations. The sequences of the primers can be found in the supplementary table S5. E. Intracellular growth assay fixed 24 hours post-invasion. The number of parasites per vacuole were determined and represented as mean +/− SD. F. Invasion assay performed using a two-color immunofluorescence. Intracellular: invaded parasites, extracellular: attached parasites. Data are represented as mean ± SD. G. Calcium ionophore-induced egress assay performed after 30 hours, expressed as a percentage of egressed vacuoles and represented as mean +/− SD. (PDF) [file ppat.1004504.s004.pdf]

Supplementary figure S5

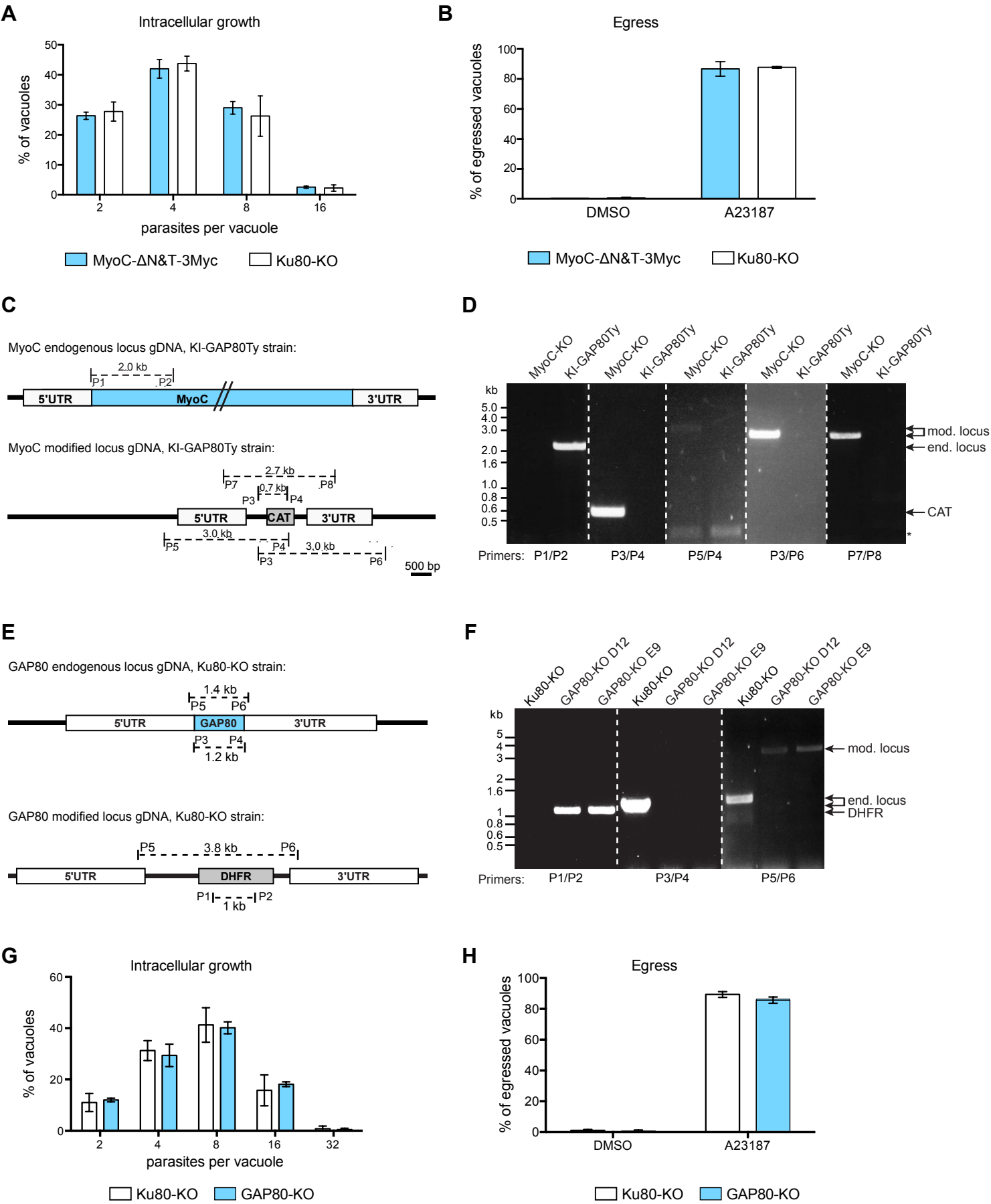

Supplement: Figure S5 — Analyses of MyoC-KO and GAP80-KO strains. A. Intracellular growth assay performed with MyoC-ΔN&T-3Myc and Ku80-KO strains and fixed after 24 hours. The number of parasites per vacuole were determined and represented as mean +/− SD. B. Calcium ionophore-induced egress assay performed with MyoC-ΔN&T-3Myc and Ku80-KO after 30 hours. Data are expressed as a percentage of egressed vacuoles and represented as mean +/− SD. C. Schematic representation of the endogenous and modified locus of MyoC in KI-GAP80Ty and MyoC-KO/KI-GAP80Ty strains, respectively. The position of the primers used to confirm the integration and the length of the PCR products are indicated. D. PCRs performed on gDNA extracted from KI-GAP80Ty and MyoC-KO/KI-GAP80Ty strains to confirm the disruption of the MyoC gene. end.: endogenous, mod.: modified, CAT: chloramphenicol acetyltransferase gene conferring resistance to chloramphenicol. The sequences of the primers can be found in the supplementary table S5. The star indicates unspecific bands. E. Schematic representation of the endogenous and modified locus of GAP80 in Ku80-KO strain. The position of the primers used to confirm the integration and the length of the PCR products are indicated. F. PCRs performed on gDNA extracted from Ku80-KO and GAP80-KO strains to confirm the disruption of the GAP80 gene. end.: endogenous, mod.: modified, DHFR: dihydrofolate reductase gene conferring resistance to pyrimethamine. The sequences of the primers can be found in the supplementary table S5. G. Intracellular growth assay performed with Ku80-KO and GAP80-KO and fixed after 24 hours. The number of parasites per vacuole were determined and represented as mean +/− SD. H. Calcium ionophore-induced egress assay performed with Ku80-KO and GAP80-KO after 30 hours, expressed as a percentage of egressed vacuoles and represented as mean +/− SD. (PDF) [file ppat.1004504.s005.pdf]

Supplementary figure S6

A

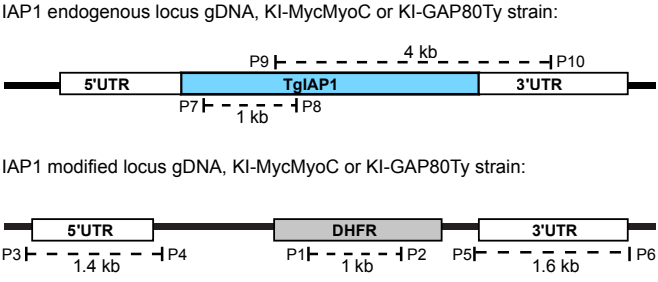

B

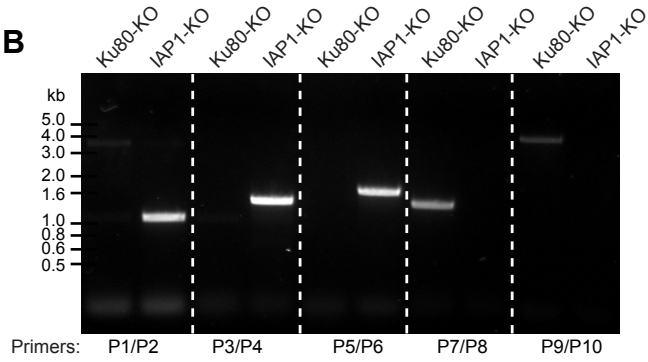

C

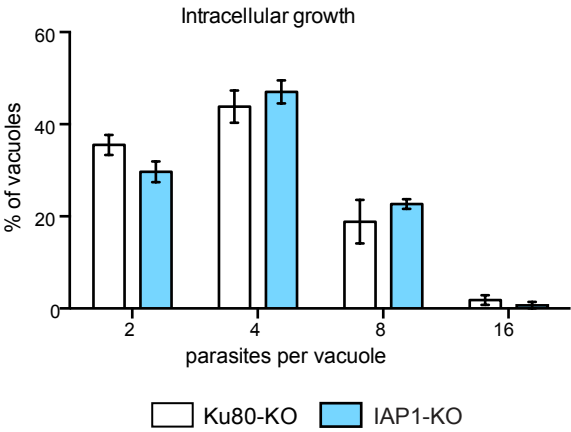

D

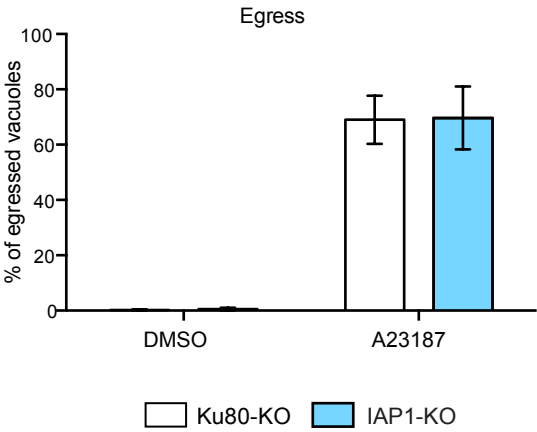

Supplement: Figure S6 — Analyses of IAP1-KO strain. A. Schematic representation of the endogenous and modified locus of IAP1 in KI-MycMyoC or in KI-GAP80Ty strains. The position of the primers used to confirm the integration and the length of the PCR products are indicated. B. PCRs performed on gDNA extracted from Ku80-KO and IAP1-KO strains to confirm the disruption of the IAP1 gene. The sequences of the primers can be found in the supplementary table S5. C. Intracellular growth assay performed with Ku80-KO and IAP1-KO and fixed after 24 hours. The number of parasites per vacuole were determined and represented as mean +/− SD. D. Calcium ionophore-induced egress assay performed with Ku80-KO and IAP1-KO after 30 hours, expressed as a percentage of egressed vacuoles and represented as mean +/− SD. (PDF) [file ppat.1004504.s006.pdf]

Supplementary figure S7

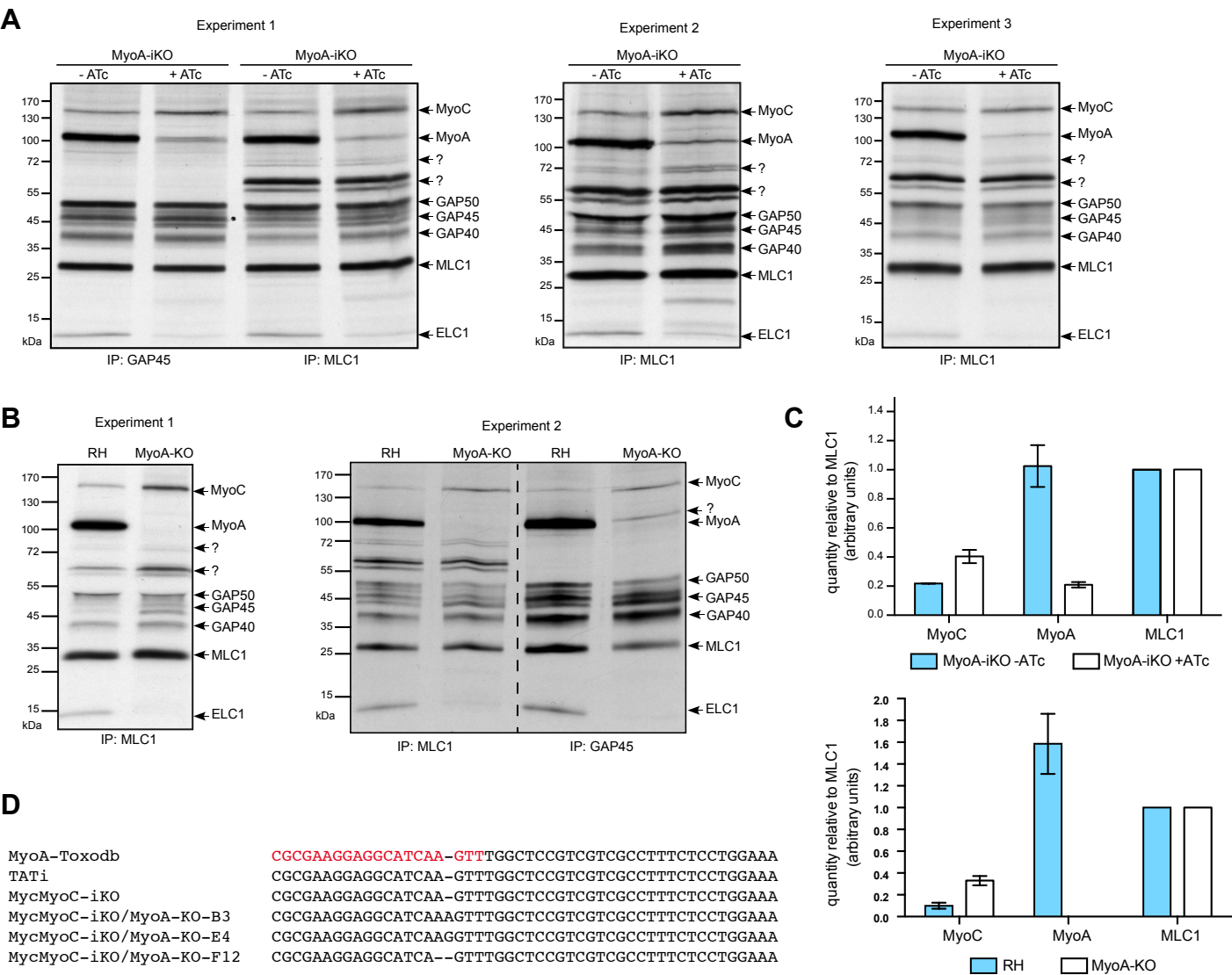

Supplement: Figure S7 — More MyoC associates with MyoA-glideosome in absence of MyoA. A–B. Autoradiograph of the bound fractions obtained after Co-IP performed with anti-MLC1 or anti-GAP45 antibodies on metabolic labeled MyoA-iKO parasites treated or not with ATc (A) or RH and MyoA-KO (B). C. Quantification of MyoA and MyoC co-immunoprecipitated with anti-MLC1 antibodies has been performed with ImageJ (see supplementary materials and methods) from 3 independent experiments performed with MyoA-iKO strain and 2 independent experiments performed with MyoA-KO. Data are represented as mean +/−. D. Sequencing of 3 independent clones to confirm the disruption of MyoA induced by CAS9. The gRNA used is written in red. (PDF) [file ppat.1004504.s007.pdf]
