# Supplementary material for: Plasticity between MyoC- and MyoA-Glideosomes: An Example of Functional Compensation in Toxoplasma gondii Invasion
Source: PLoS Pathog. 2014 Nov 13;10(11):e1004504. doi: 10.1371/journal.ppat.1004504 (PMC4231161; doi:10.1371/journal.ppat.1004504)
Supplement: Table S1 — Identification of MyoC and GAP80 by mass spectrometry after co-IP performed with anti-Ty antibodies on parasites stably expressing TgMLC1Ty. (PDF) [file ppat.1004504.s008.pdf]

Table S1. Identification of MyoC and GAP80 by mass spectrometry after co-IP performed with anti-Ty antibodies on parasites stably expressing TgMLC1Ty.

Protein identification	Number of unique peptides	List of peptides
TGME49_255190<br>MyoB/C	31 (33% coverage)	AATNFTCWTADCPAVK<br>ALFEGIEIEAGK<br>DLGNTTDAWISTYR<br>DVSYLIGMLFQR (specific to MyoC)<br>EALLSGMERPR<br>EVAQLLLEQSGIPESSWVIGK<br>FMMLDVSSHR<br>GIQHGSISNFLLEK<br>HLEPDSINISPEER<br>HTIADIEYTCEGMLEK<br>LEPSGFFLESR<br>LYQAEGVPTEALEYTDNLALVGALCGK<br>NASKPEMLPPHVFK<br>NDSFFALLEDACLGIR<br>NFSEFCSHFR<br>NPCVVVK<br>NQSIIVSGESGAGKTEATK<br>RHLEPDSINISPEER<br>RLEPSGFFLESR<br>RLQNICTVIR<br>RPSHVCMEEAYHVWR<br>SIIYTTAEPLLVAINPFK<br>SYHIFYQLLK<br>TMVFVKPDAAK<br>TTIQDTIMAGNPILEAFGNAK<br>VGPQVIEGVR<br>VIHFLTR<br>VLNQLFSLSILEALQLR<br>VVSQEANER<br>WLDLGLVNSDR
TGME49_246940<br>GAP80	7 (27% coverage)	AAQEIPAKQPEEEEEEETQIPLR<br>AEDLGEAPLSRPASIPVEMTTR<br>DFDFSDIPVLQ<br>EASQEVQLVNRDPVPVSPPPK<br>LAELETLQTLAEVTYYR<br>NPFGFCFGGGK<br>TRAEDLGEAPLSRPASIPVEMTTR

Accession numbers are from EupathDB (Aurrecoechea et al., 2007).
