# Supplementary material for: Plasticity between MyoC- and MyoA-Glideosomes: An Example of Functional Compensation in Toxoplasma gondii Invasion
Source: PLoS Pathog. 2014 Nov 13;10(11):e1004504. doi: 10.1371/journal.ppat.1004504 (PMC4231161; doi:10.1371/journal.ppat.1004504)
Supplement: Table S2 — List of the proteins identified by mass spectrometry from co-IP performed with anti-Myc antibodies on parasites stably expressing MycGFPCtGAP80 as a second copy. (PDF) [file ppat.1004504.s009.pdf]

Table S2. List of the 15 proteins identified by mass spectrometry from the 2 bands cut after co-IP performed with anti-Myc antibodies on parasites stably expressing MycGFPCtGAP80 as a second copy. As a control, the same co-IP has been performed with MycGFPCtGAP70 and bands around the same size have been cut out and analysed (see supplementary Figure S3A).

Band	Protein Identification<br>Protein name	Present in the control	MW (kDa)	Nb of unique peptides (coverage)	Localization
1	TGME49_220090<br>Putative uncharacterized protein	yes	28	1 (6%)	n.d.
	TGME49_263300<br>Porin, putative	no	31	3 (16%)	n.d.
	TGME49_283510<br>Putative uncharacterized protein	no	35	7 (28%)	Posterior IMC and basal ring
	TGME49_286720<br>Heat shock protein 28, HSP28	no	31	2 (12%)	n.d.
	TGME49_289690<br>Glyceraldehyde-3-phosphate dehydrogenase, GAPDH1	no	37	4 (19%)	n.d.
	TGME49_294670<br>Eukaryotic translation initiation factor 3, putative	no	32	2 (12%)	n.d.
	TGME49_308840<br>SRS domain-containing protein, SRS3	no	38	2 (6%)	n.d.
	TGME49_310420<br>Putative uncharacterized protein	no	14	2 (18%)	Pellicle
2	TGME49_220090<br>Putative uncharacterized protein	yes	28	3 (19%)	n.d.
	TGME49_232710<br>40S ribosomal protein S3a, RPS3A	yes	29	3 (19%)	n.d.
	TGME49_233460<br>Surface antigen protein 1, SAG1	yes	35	4 (16%)	Surface
	TGME49_257680<br>Myosin light chain, MLC1	no	24	2 (14%)	Pellicle
	TGME49_263090<br>14-3-3 protein, putative	yes	37	3 (11%)	Basal cup and DC (Lorestani et al., 2012)
	TGME49_286720<br>Heat shock protein 28, HSP28	yes	31	10 (46%)	n.d.
	TGME49_313400<br>DnaJ domain-containing protein	yes	44	4 (13%)	n.d.
	TGME49_219320<br>Gliding-associated protein 50, GAP50	no	47	2 (8%)	IMC
	TGGT1_053770 Conserved hypothetical protein	no	48	2 (5%)	Dense granule

Band 1 corresponds to the band cut out around 40 kDa and band 2 to the one cut out above 35 kDa. In green are the proteins investigated, in salmon are two other proteins of the complex, GAP50 and MLC1. Accession numbers are from EupathDB (Aurrecoechea et al., 2007), MW: molecular weight, nb: number, n.d. : not determined.
