# Supplementary material for: Plasticity between MyoC- and MyoA-Glideosomes: An Example of Functional Compensation in Toxoplasma gondii Invasion
Source: PLoS Pathog. 2014 Nov 13;10(11):e1004504. doi: 10.1371/journal.ppat.1004504 (PMC4231161; doi:10.1371/journal.ppat.1004504)
Supplement: Table S3 — Identification of IAP1 by mass spectrometry after co-IP performed with anti-Myc antibodies on parasites stably expressing MycGFPCtGAP80. (PDF) [file ppat.1004504.s010.pdf]

Table S3. Identification of IAP1 by mass spectrometry after co-IP performed with anti-Myc antibodies on parasites stably expressing MycGFPCtGAP80.

Protein identification	Number of unique peptides	List of peptides
TGME49_283510<br>IAP1	7 (28% coverage)	EAVAGFSIER<br>FLTTIDLR<br>GDDAVSHSSITGDCDLNR<br>LQALVKEFAK<br>LSFDIAR<br>QGTTPLELQSTSAKPPYR<br>SKAFDGDGSVPPPMSPAK

Accession numbers are from EupathDB (Aurrecoechea et al., 2007).
