# Supplementary material for: Plasticity between MyoC- and MyoA-Glideosomes: An Example of Functional Compensation in Toxoplasma gondii Invasion
Source: PLoS Pathog. 2014 Nov 13;10(11):e1004504. doi: 10.1371/journal.ppat.1004504 (PMC4231161; doi:10.1371/journal.ppat.1004504)
Supplement: Table S4 — Oligonucleotide primers used in this study for cloning. (PDF) [file ppat.1004504.s011.pdf]

Table S4. Oligonucleotide primers used in this study for cloning. The restriction sites are underlined. F: forward, R: reverse.

Name	Sequence 5'-3'
GAP80-1F	CCGCCTGCAGGGGCTCGAAGACTTCAGGCGAAACTCG
GAP80-2R	GGCTTAATTAACACTGAAGAACTGGGATGTCTGAGAAG
GAP80-3F	CCGGAATTCAAAATGGGACGCATGAAGAATCCGTTCGG
GAP80-4R	GGCATGCATGTCATCGTCTGAAAATCGTCCCC
GAP80-5F	CCCAAGCTTCAATTGGCGCTGAAGCCTCGTTCGATAGCC
GAP80-6R	CCCAAGCTTAGTGCCGGCAGAGATGGATGACG
GAP80-7F	CGGGCGGCCGCGGAATCAGAGAGACTGCG
GAP80-8R	CGGGCGGCCGCAATTGCAGGCTCTTCTCCGCGGCTC
GAP70-1F	GGGGTACCTATCGTCCTTCCAGATGC
GAP70-2R	ATGCATACGTCGCTGGCAGGTGTTACC
ELC1-1F	CCGGGTACCCCAGACGAAATCAAGGCAC
ELC1-2R	GGCATGCATGTTTCAGCAGCATCTTGACAAAGTTGG
IAP1-1F	CCGGAATTCAGCCGTTCCTTGAGGACAAG
IAP1-2R	TGCATGCATGCTGCCGCTTATCTTTTCTGGC
IAP1-3F	GGCGAATTCATGGACGCCGCCGGACTGGCTGCCGATGATGATCGGGACGTGTGG
IAP1-4F	GGGGTACCTGAACGCGAGTGTGGGCCAC
IAP1-5F	TACTTCCAATCCAATTTAATGCAAACATGTCTCCAATGGGAGTGTCG
IAP1-6R	TCCTCCACTTCCAATTTTAGCCTTTGCCGAGGTCGACTGTAGTTC
IAP1-7F	GCCGGGCCCTGCAGGAGATCAACTTCTAAAGCCAGAAAATGC
IAP1-8R	CCGGGGCCCTGTTTGAAACCTGAAGGTCAAC
IAP1-9F	GCTCTAGAACAAGCGAAAACGTGCCCCG
IAP1-10R	GCGGCCGCCTGCAGGCTCAGCTATGATACGATGAACG
ILP1-1F	GGGGTACCGGACGCGCCCGAGAGTAGAG
ILP1-2R	GGCATGCATGGCAGCACGCTCGACTTGGGTGTTCC
MyoC-1F	GGGGTACCGTACGCGTATGTGTCTGTAGG
MyoC-2R	CCATGCATGCGGTCTATCCGGCGCACAG
MyoC-3F	CTGCAGATGGAGCGCAAACAAACCCAGATGATAC
MyoC-4R	GGATCCGTTTAAACGACAGCGACAACACAGACGTCTTGG
MyoC-5F	GGGCCCGTTTAAACGCTGTACGTCACCTGCACAGCG
MyoC-6R	GGGCCCCGCTTTCTCGTCGGAGGACAG
MyoC-7F	TACTTCCAATCCAATTTAATGCGTGCACTTGTGGATGGTGTTTG
MyoC-8R	TCCTCCACTTCCAATTTTAGCCCACGACGACTCCGGAATTCCTGATTG
MyoC-9F	GGCCGGATCCCTGCTCTAGCAGAACAGGC
MyoC-10R	GCTTGCGGCCGCAGCTGCATCTGGAGGC
MyoC-11F	GCGAGGACTGTGATGAGTATGC
MyoC-12R	GCAGACCTGTAGGATCTTGTTAG
MyoA-gRNA	GCGCGAAGGAGGCATCAAGTTGTTTTAGAGCTAGAAATAGC
MyoC-gRNA	GCCAGCACGCGGGACGTTTAGTTTTAGAGCTAGAAATAGC
gRNA-rev	AACTTGACATCCCCATTTAC
IMC1-1F	CGGGATCCGAGAAGGTGGTTGAGGTACCTG
IMC1-2R	CCGCTCGAGTTAGCACTGGCATCGGCACAC
