# Supplementary material for: Plasticity between MyoC- and MyoA-Glideosomes: An Example of Functional Compensation in Toxoplasma gondii Invasion
Source: PLoS Pathog. 2014 Nov 13;10(11):e1004504. doi: 10.1371/journal.ppat.1004504 (PMC4231161; doi:10.1371/journal.ppat.1004504)
Supplement: Table S5 — Oligonucleotide primers used in this study for PCR analyses. (PDF) [file ppat.1004504.s012.pdf]

Table S5. Oligonucleotide primers used in this study for PCR analyses. P: primers used in supplementary figures S4, S5 and S6.

Figure	Name	Sequence 5'-3'
S4C	MyoC-P1	CTGCAGATGGAGCGCAAACAAACCCAGATGATAC
	MyoC-P2	GGATCCGTTTAAACGACAGCGACAACACAGACGTCTTGG
	MyoC-P5	GCGAGGACTGTGATGAGTATGC
	MyoC-P6	GCAGACCTGTAGGATCTTGTTAG
	MyoC- P7	GCACACGGTAGATGACCTTCTG
	MyoC- P8	CCATGCATGCGGTCTATCCGGCGCACAG
	CAT-P3	CCGGGCATGCAGGAGAAAAAAATCACTGGA
	CAT-P4	GCCCCGCCCTGCCACTCATCGC
S4E	GAP80-P3	CCGGAATTCAAAATGGGACGCATGAAGAATCCGTTCGG
	GAP80-P4	GGCTTAATTAACACTGAAGAACTGGGATGTCTGAGAAG
	GAP80-P5	CTCTGGAGACAAAGTAACAGTCCTG
	GAP80-P6	GCGTTTCACTCTCTGGTCATGACGAC
	DHFR-P1	GGAAATTTGCAAAGACGGGCGACTCTGG
	DHFR-P2	TTGTCCCAGATCTTCACGCCCTTCTCAG
S5A	IAP1-P3	CGCTGGCCAGCTTGTCTCCG
	IAP1-P6	CTTGCGTAGGCGCCCTACTAC
	IAP1-P7	CCGGAATTCAGCCGTTCCTTGAGGACAAG
	IAP1-P8	GAACTACAGTCGACCTCGGCAAAGGCTAAAATTGGAAGTGGAGGA
	IAP1-P9	GGCATGCATGTGGCTGCGTCGTTGCCCCAG
	IAP1-P10	CCTTAATTAATCACTGCCGCTTATCTTTTCTGGC
	DHFR-P1	GGAAATTTGCAAAGACGGGCGACTCTGG
	DHFR-P2	TTGTCCCAGATCTTCACGCCCTTCTCAG
	DHFR-P4	GAGGACGACTCACGGGATTTACAGCC
	DHFR-P5	GCCCACGACAGCAGACAACTTTCC
S7A S7B	MyoC-P1	CCCTGCTGGGTCGCACG
	MyoC-P2	GCAAGGTTATCCGTGTACTCG
	MyoC-P3	CCGTAATGTCTCAGCGGCATC
	CAT-P4	ACACAAGGTGATTGTGTAACACCG
	Myc-P5	AGCAGAAGCTCATCTCCGAGGAG
