# Supplementary material for: Plasticity between MyoC- and MyoA-Glideosomes: An Example of Functional Compensation in Toxoplasma gondii Invasion
Source: PLoS Pathog. 2014 Nov 13;10(11):e1004504. doi: 10.1371/journal.ppat.1004504 (PMC4231161; doi:10.1371/journal.ppat.1004504)
Supplement: File S1 — Supplementary materials and methods. (DOCX) [file ppat.1004504.s013.docx]

**Supplementary materials and methods**

***Secondary structure prediction***

*In silico* predictions of the coiled-coil domains has been performed using COILS [[1](#_ENREF_1)]. Prediction of the secondary structures has been performed on the Network Protein Sequence Analysis server (NPS@, npsa-pbil.ibcp.fr) using the consensus secondary structure prediction of the following methods: DPM [[2](#_ENREF_2)],  DSC [[3](#_ENREF_3)], GOR IV [[4](#_ENREF_4)], HNN [[5](#_ENREF_5)],  PHD [[6](#_ENREF_6),[7](#_ENREF_7)], PREDATOR [[8](#_ENREF_8)], SIMPA96 [[9](#_ENREF_9),[10](#_ENREF_10)] and SOPMA [[11](#_ENREF_11)].

***Co-IP quantifications***

Densitometric analysis of the autoradiographies was carried out using the gel analysis module of ImageJ software. The intensity of the bands was normalized to the intensity of MLC1 band that was arbitrarily set to 1.

***Time-lapse video microscopy***

For the monitored induced egress assay, freshly egressed parasites were allowed to invade host cells grown in 35 mm glass dishes. 30 h later, the complete medium was changed for 1 mL of DMEM and 1 mL of DMEM containing 6 μM of the Ca^2+^ ionophore A23187 from *Streptomyces* *chartreusensis* (calbiochem) was added just before recording. Movies were recorded with a Zeiss axiovert 200M microscope with a objectif apochromat 63x/1.4 DIC during 10-15 minutes with one picture taken every 500 ms. The files were then processed using ImageJ software.

**References**

1. Lupas A, Van Dyke M, Stock J (1991) Predicting coiled coils from protein sequences. Science 252: 1162-1164.

2. Deleage G, Roux B (1987) An algorithm for protein secondary structure prediction based on class prediction. Protein Eng 1: 289-294.

3. King RD, Sternberg MJ (1996) Identification and application of the concepts important for accurate and reliable protein secondary structure prediction. Protein Sci 5: 2298-2310.

4. Garnier J, Gibrat JF, Robson B (1996) GOR method for predicting protein secondary structure from amino acid sequence. Methods Enzymol 266: 540-553.

5. Guermeur Y, Geourjon C, Gallinari P, Deleage G (1999) Improved performance in protein secondary structure prediction by inhomogeneous score combination. Bioinformatics 15: 413-421.

6. Rost B, Sander C (1993) Prediction of protein secondary structure at better than 70% accuracy. J Mol Biol 232: 584-599.

7. Rost B, Sander C (1994) Combining evolutionary information and neural networks to predict protein secondary structure. Proteins 19: 55-72.

8. Frishman D, Argos P (1996) Incorporation of non-local interactions in protein secondary structure prediction from the amino acid sequence. Protein Eng 9: 133-142.

9. Levin JM (1997) Exploring the limits of nearest neighbour secondary structure prediction. Protein Eng 10: 771-776.

10. Levin JM, Robson B, Garnier J (1986) An algorithm for secondary structure determination in proteins based on sequence similarity. FEBS Lett 205: 303-308.

11. Geourjon C, Deleage G (1995) SOPMA: significant improvements in protein secondary structure prediction by consensus prediction from multiple alignments. Comput Appl Biosci 11: 681-684.
